# Reprogramming of endothelial gene expression by tamoxifen inhibits angiogenesis and ERα-negative tumor growth

**DOI:** 10.7150/thno.87306

**Published:** 2024-01-01

**Authors:** Chanaëlle Fébrissy, Marine Adlanmerini, Christel Péqueux, Frédéric Boudou, Mélissa Buscato, Adrien Gargaros, Silveric Gilardi-Bresson, Khrystyna Boriak, Henrik Laurell, Coralie Fontaine, Benita S. Katzenellenbogen, John A. Katzenellenbogen, Julie Guillermet-Guibert, Jean-François Arnal, Raphaël Metivier, Françoise Lenfant

**Affiliations:** 1 INSERM U1297, Institut des Maladies Métaboliques et Cardiovasculaires, Université de Toulouse, BP 84225, 31 432 Toulouse cedex 04, France.; 2 Laboratoire de Biologie des Tumeurs et du Développement, GIGA-Cancer, Université de Liège, B23, Liège, Belgium.; 3 Departments of Molecular and Integrative Physiology and Chemistry, University of Illinois, Urbana, Illinois, USA.; 4 INSERM U1037, CRCT, Oncopole- 31 037 Toulouse cedex, France.; 5 Institut de Génétique De Rennes (IGDR). UMR 6290 CNRS-Université de Rennes, ERL INSERM U1305. CS 74205- 35042 Rennes Cedex, France.

**Keywords:** Tamoxifen, Angiogenesis, Estrogen Receptor ERα, Endothelial cells, Tumor growth

## Abstract

**Rationale**: 17β-estradiol (E2) can directly promote the growth of ERα-negative cancer cells through activation of endothelial ERα in the tumor microenvironment, thereby increasing a normalized tumor angiogenesis. ERα acts as a transcription factor through its nuclear transcriptional AF-1 and AF-2 transactivation functions, but membrane ERα plays also an important role in endothelium. The present study aims to decipher the respective roles of these two pathways in ERα-negative tumor growth. Moreover, we delineate the actions of tamoxifen, a Selective Estrogen Receptor Modulator (SERM) in ERα-negative tumors growth and angiogenesis, since we recently demonstrated that tamoxifen impacts vasculature functions through complex modulation of ERα activity.

**Methods:** ERα-negative B16K1 cancer cells were grafted into immunocompetent mice mutated for ERα-subfunctions and tumor growths were analyzed in these different models in response to E2 and/or tamoxifen treatment. Furthermore, RNA sequencings were analyzed in endothelial cells in response to these different treatments and validated by RT-qPCR and western blot.

**Results:** We demonstrate that both nuclear and membrane ERα actions are required for the pro-tumoral effects of E2, while tamoxifen totally abrogates the E2-induced *in vivo* tumor growth, through inhibition of angiogenesis but promotion of vessel normalization. RNA sequencing indicates that tamoxifen inhibits the E2-induced genes, but also initiates a specific transcriptional program that especially regulates angiogenic genes and differentially regulates glycolysis, oxidative phosphorylation and inflammatory responses in endothelial cells.

**Conclusion:** These findings provide evidence that tamoxifen specifically inhibits angiogenesis through a reprogramming of endothelial gene expression via regulation of some transcription factors, that could open new promising strategies to manage cancer therapies affecting the tumor microenvironment of ERα-negative tumors.

## Introduction

Estrogen receptor α (ERα) is a key element in the diagnosis and treatment of breast cancers. Indeed, its expression by cancer cells determines the use of hormonal treatments, such as tamoxifen or aromatase inhibitors, blocking the activity or the synthesis of estrogens, respectively 1, 2. Tamoxifen is a selective ER modulator (SERM) and is the most frequently prescribed drug in pre-menopausal women. It effectively inhibits estrogen-stimulated growth of breast cancer cells by competitively binding and blocking ERα. Although ERα-negative patients are considered to be non-responders, it has been noted that 5% to 10% of patients with ERα-negative tumors do benefit from adjuvant therapy, reducing the risk of recurrent disease by 9% (*P* = 0.03) and the mortality risk by 6% (not statistically significant) 3, 4. An increasing number of data argues that stromal ERα in the tumor microenvironment also contributes to malignant development and progression of various cancers 2. Indeed, ovariectomy decreases recurrence risk and mortality of breast cancer classified as ERα-positive and also as ERα-negative 5. Several studies have also delineated that estrogen action on the stromal tumor environment enhances tumor growth 6-9. Previously, we demonstrated that 17β-estradiol (E2) accelerates the growth of ERα-negative tumors (B16K1 melanoma, LLC lung and 4T1 mammary cancer cells) through the development of a vascular supply by activating stromal ERα that normalizes tumor angiogenesis 10. Interestingly, tamoxifen has shown some efficacy in reducing ERα-negative tumor growth by inhibiting angiogenesis in an ER-negative fibrosarcoma model 11 and in female mice with ER-negative lung cancer 12. Although tamoxifen has been used successfully for 40 years in medicine, understanding the mechanisms of action of tamoxifen on tumor microenvironment needs particular investigation. In this context, we shed light on the molecular mechanisms activated by E2 to induce ERα-negative tumor growth and by tamoxifen to block the E2-induced ERα-negative tumor growth.

ERα protein is a member of the nuclear receptor (NR) family of transcriptional regulators. ERα modulates gene transcription in a cell and tissue-specific manner through their two activation functions (AFs), AF1 and AF2, which are necessary for the recruitment of transcriptional machinery by ERα 13. Several studies have shown that ERα contributes to angiogenesis principally through the transcriptional regulation of genes encoding angiogenic factors such as Fibroblast Growth Factor-2 12, 14, Vascular Endothelial Growth Factor (VEGF) and their receptors VEGFRs 15, 16. These ERα-dependent transcriptional regulations (so-called nuclear actions) are essential for E2-induced angiogenesis. Nevertheless, the direct involvement of AF1 or AF2 functions of ERα in E2-induced angiogenesis has not been investigated yet. Moreover, additional evidences suggest that the rapid actions of estrogen could also be relevant. These rapid effects have been attributed to “Membrane-Initiated Steroid Signaling” (MISS) but do not exclude subsequent potential transcription activity 17, 18. Membrane ERα is crucial for vascular effects of estrogen 19-21. Using mice deficient for membrane-anchored ERα, through the mutation of the palmitoylation site Cys451 of ERα (ERα-C451A mice) in which the E2-induced endothelial cell migration is lost, we demonstrated that membrane ERα signaling is necessary and even sufficient to mediate E2-induced vasodilatation and E2-induced re-endothelialization 22. Some studies demonstrated a direct involvement of MISS signaling in angiogenesis acting on endothelial cell migration, largely through rapid signaling pathways including eNOS, PI3K/Akt and Erk 5, 23. Nevertheless, the role of membrane ERα signaling in tumor angiogenesis *in vivo* has never been investigated yet.

In the present study, using the model of ERα-negative cancer cells B16K1 grafted subcutaneously into syngeneic ovariectomized immunocompetent mice, the combination of genetic and pharmacological approaches revealed that both nuclear (AF1/AF2-mediated) and membrane actions of ERα are required for the pro-tumoral effects of E2 while the sole activation of membrane signaling of ERα is not sufficient. More importantly, this work provides evidences that tamoxifen alone has no impact on the growth of ERα-negative tumors, but that it antagonizes E2-induced tumor growth by inhibiting angiogenesis in parallel to vessel normalization. Finally, RNA sequencing indicates that 4-hydroxytamoxifen (4-OHT), the metabolite of tamoxifen, not only antagonizes the E2-induced genes but elicits a specific transcriptional program in endothelial cells that impacts both angiogenesis and endothelial metabolism (glycolysis and oxidative phosphorylation) through the regulation of key transcription factors.

## Materials and Methods

### Animals

Female C57BL/6J mice were purchased from Charles River. Mice deleted for ERα (ERα^-/-^), ERα-AF1 (ERα-AF1^0^), ERα-AF2 (ERα-AF2^0^) and mutated for C451A (C451A-ERα) have been previously described 22, 24. All procedures were performed in accordance with the guidelines established by the National Institute of Medical Research and were approved by the local Ethical Committee of Animal Care and French Ministry of Research (Protocols# CEA-122-DAP-2015-15 and CEA-122-2022120814504579). Mice were ovariectomized at 4 weeks of age. Two weeks after ovariectomy, mice were treated with E2 (0.01 mg in 60-day-release pellets, Innovative Research of America), Tamoxifen (1.5-4 mg in 60-day-release pellets, Innovative Research of America), EDC (estradiol dendrimer conjugate, 240 µg⋅kg^-1^⋅d^-1^, kindly provided by J. Katzenellenbogen, using Alzet minipumps) or empty dendrimer at a rate identical to that delivered with EDC as a control 25, 26.

### Cell culture

B16K1 is a genetically modified cell line obtained from B16F10 cells, which stably express MHC-I molecule H-2Kb 10. B16K1 was last authenticated in February 2012 by Leibniz-Institut DSMZ GmbH. B16K1 cell were cultivated in Dulbecco's modified Eagle's medium (DMEM) supplemented with 10% FBS. Immortalized human aortic endothelial cells (TeloHAEC, ATCC-CRL-4052) were a gift from A.Salvayre-Negre (INSERM U1297, I2MC, Toulouse) and cultivated in Endothelial Cell Growth medium (Promocell C-22010) with 1x penicillin/streptomycin (Sigma). B16K1 and TeloHAEC cells were cultivated at 37°C, 5% CO_2_.

### *In vivo* tumor models and quantification of vessel density and vessel normalization

Tumor B16K1 cells (4x10^5^ cells resuspended in PBS) were injected subcutaneously to both flanks of wild type (WT) or transgenic C57BL/6J mice. Tumor volume was measured every 2 to 3 days with digital caliper and calculated as V (mm^3^) = π x ((width)2 x length)/6. Before euthanasia, mice were anesthetized and perfused by intravenous injection of fluorescein isothiocyanate (FITC)- conjugated lectin (100 μg in 100 μl 0.9% NaCl, #FL-1171, Vector Laboratories, CA, USA) that was allowed to circulate for 5 minutes. The tumor vasculature was then fixed by intracardiac perfusion of 4% PFA and then embedded in OCT. Following lectin-FITC injection, fluorescence and photostability of FITC-conjugated lectin was enhanced with the AlexaFluor 488 Signal-Amplification Kit performed on 6 μm-thick tumor sections (Molecular Probes). Immunostainings were performed using primary antibodies: anti-CD31 (MEC13.3, BD Pharmingen), anti-αSMA conjugated-Cy3 (1A4, Sigma). Then sections were incubated with the conjugated secondary antibodies AlexaFluor 647 (Abcam) and the nuclei were counterstained with DAPI (Invitrogen, Life Technologies). The samples were then mounted with fluoromount-G (Southern Biotech), and acquired using a confocal microscope (Zeiss LSM900). The images were further processed using ImageJ 1.53v.

### Aortic rings assay

Mouse aortic ring assay was performed as previously described 27 on WT or transgenic mice ovariectomized and treated as previously described during 2-3 weeks before the procedure. Briefly, 2 mm aortic rings were cultured in collagen gel (1.5 mg/ml). The aortic rings were stimulated with 2.5% of autologous mice serum in MCDB131 medium (Gibco, 10372). The explants were cultured for 9 days at 37°C and 5% CO_2_.

### RNA-sequencing and differential gene expression analysis

Human endothelial TeloHAEC cells transduced with hERα-WT (TeloHAEC-hERα-WT 28) or not were cultured in Phenol-Red free Endothelial Cell growth medium (Promocell) with Penicillin/streptomycin (Sigma) and charcoaled-treated fetal calf serum, then treated for 6 hours with DMSO vehicle control, E2 (1nM) or 4-OHT (100 nM) alone or in combination with E2 before collection. Total RNA from TeloHAEC-hERα-WT was extracted through the phenol-chloroform method using TRIzol (Ambion) reagent. Sequencing was performed in paired-end (2x150 bp) on an Illumina NovaSeq sequencer at the Genewiz/Azenta commercial sequencing facility (Leipzig, Germany). All submitted samples had an RNA integrity number (RIN) >8. Sequence reads were trimmed to remove possible adapter sequences and nucleotides with poor quality using Trimmomatic v.0.36. Trimmed reads were mapped to the Homo sapiens GRCh38 reference genome available on ENSEMBL using the STAR aligner v.2.5.2b. Unique gene hit counts were calculated by using feature Counts from the Subread package v.1.5.2, with only unique reads that fell within exon regions being counted. Fastq files are accessible through the GEO portal 29 under the GSE219159 accession number. Differential gene expression analysis was performed using DESeq2 suite 30. Following the removal of genes with no detected expression, weakly expressed genes were filtered upon a threshold value of expression calculated by using the HTSfilter package 31. Genes were then declared as differentially regulated when their absolute fold change (FC) was > 1.5 with a BH adjusted *p*-value < 0.05. Functional annotations were made by interrogating MSigDB hallmarks 32 through the R cluster Profiler package 33 using enricher function for over-representation analysis (ORA) and the integrated GSEA function for global gene set enrichment analysis 34. Dot-plots of enriched Hallmarks terms as well as GSEA enrichment plots with ranked list metrics were generated using the enrich plot R library 35.

### Transcription factors binding motifs analysis

Enrichment for transcription factors binding motifs was assessed within a -10/+10 kb window centered on the TSS of genes regulated either by E2, 4-OHT or both ligands. To do so, we used the RcisTarget R package 34 to interrogate the presence of TF motifs or the presence of an actual TF binding site (TFBS) as determined from Encode knowledge in these windows (hg38 genome annotations). We retained associations having a Normalized Enrichment Score (NES) ≥ 3. Resulting motifs-to-genes and TFBS-to-genes-associations were then merged and sorted in order to generate a Transcription Factor-to-genes association file which was then transformed in incidence matrices using the Sort_Incidence_matrix function of the RcisTarget package and subsequently sorted in edges and nodes files compatible with visualization under Cytoscape v3.9.1 35.

### Gene Expression Analysis by RT-qPCR

Total RNA from TeloHAEC cells transduced or not was prepared using TRIzol. One thousand nanograms of RNA was reverse transcribed at 25 °C for 10 minutes and then at 37 °C for 2 hours in 20 µL final volume using the High-Capacity cDNA reverse transcriptase kit (Applied Biosystems). For gene expression, qPCR was performed using SsoFast Eva Green Supermix (Bio-Rad) on the Quant Studio 5 instrument (Applied Biosystems). Primers were validated by testing the PCR efficiency using standard curves (95% < efficiency < 105%) and are listed on **Table S1**. Gene expression was quantified using the comparative Ct (threshold cycle) method; Hypoxanthine guanine phosphoribosyl transferase (*HPRT*) was used as reference.

### Western Blot

Cells were harvested on ice, washed twice with cold PBS, collected, lysed in 50 mmol/L Tris-HCl, pH 7.5, 150 mmol/L NaCl, 1 mmol/L EDTA, 1% TritonX-100 (X100; Sigma) supplemented with protease and phosphatase inhibitors (sodium orthovanadate [S6508; Sigma], 1 mmol/L DTT, 2 mmol/L NaF [S1504; Sigma and complete Mini Protease Inhibitor Cocktail [ROCHE]). Western blotting was conducted using standard methods and as previously described 36 with the following primary antibodies: NR2F2 (ab41859, 1/1000, Abcam), NR2F6 (ab137496, 1/1000, Abcam), SMAD1 (#9743, 1/1000, Cell signaling). Protein ladder was the Lonza ProSieve™ QuadColor™ Protein Mark.

### Statistics

Results are expressed as the mean ± SEM. To test the effect of treatments or genotypes, one-way ANOVA or Kruskal-Wallis test was performed. To test the interaction between treatments and genotypes, a two-way ANOVA was carried out. When an interaction was observed between two variables, the effect of treatment was studied in each genotype using the Bonferroni post hoc test. A value of* P* < 0.05 was considered as statistically significant (*: *P* < 0.05; **: *P* < 0.01; ***: *P* < 0.001). All statistics are presented on **Table S3**.

## Results

### Both nuclear and membrane ERα signaling are necessary to promote ERα-negative tumor growth while membrane ERα alone is not sufficient

**Figure 1:**
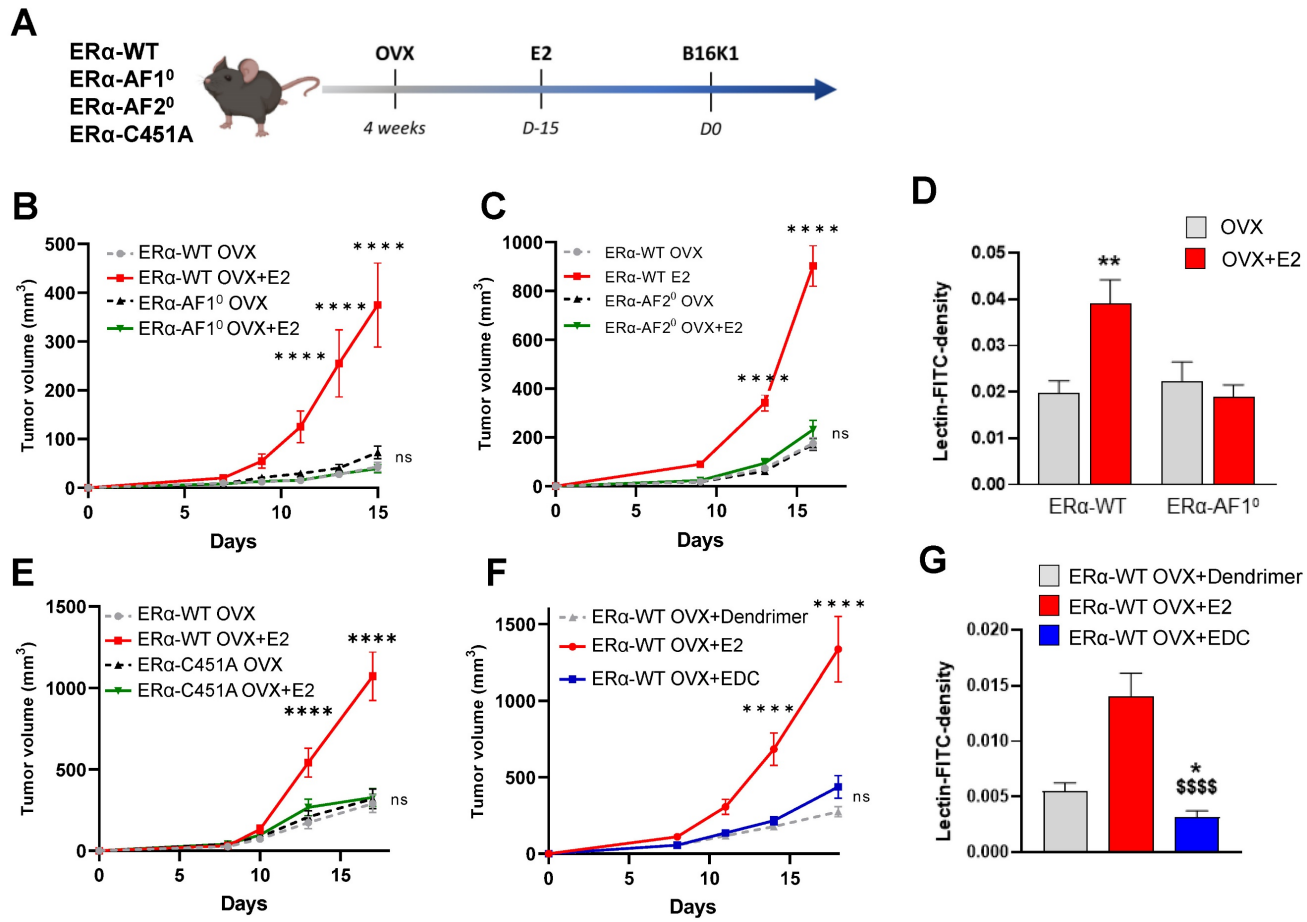
** Both nuclear and membrane ERα signaling are necessary for E2-induced growth of ERα-negative tumors and angiogenesis.** (**A**) Schematic representation of the methodology used to determine the *in vivo* growth curves of B16K1 cells injected to ovariectomized ERα-AF1^0^ (**B**), ERα-AF2^0^ (**C**) or ERα-C451A (**E**) mice untreated (OVX) or treated with E2 (E2). Their control littermates were injected as ERα-WT mice. (**D**) Perfused vessel density as measured by lectin-FITC density tumors harvested from ovariectomized ERα-AF1^0^ treated or not with E2 compared to their control littermates. Percentage of perfused vessels, n = 5 to 8 tumors. All results are mean ± SEM. Significance was determined by Multiple Mann-Whitney test followed by Benjamini, Kriedger and Yekutieli post-test: **P < 0.01, E2 *versus* OVX. (**F**) *In vivo* growth curves of B16K1 injected in ovariectomized mice treated with either Dendrimer control, E2 or EDC. n = 10-16 tumors/group. All results are mean ± SEM. Statistically relevant differences were estimated by a two-way ANOVA test followed by Bonferroni multiple comparison tests. ****: *P* < 0.0001, significant changes between OVX+E2 and all the other genotypes; ns: non-significant changes between both OVX and E2-treated ERα-AF1^0^, ERα-AF2^0^ or ERα-C451A mice and OVX+Dendrimer and EDC treated ERα-WT. (**G**) Perfused vessel density as measured by lectin-FITC density in B16K1 tumors grown in mice treated with control dendrimer (Dend), E2 or Estrogen Dendrimer Conjugate (EDC). Significance was determined by Multiple Kruskal-Wallis test followed by Dunn's post-test and illustrated as follows: significant change between dendrimer and EDC groups, (* P < 0.05); significant difference between EDC and E2; ($$$$, *P* < 0.0001). n = 21-40 slides quantified/group.

We first determined the respective roles of nuclear and membrane ERα signaling in ERα-negative tumors. To do so, we injected the ERα-negative cancer cells B16K1 to immunocompetent ovariectomized mice deleted either for ERα-AF1 (ERα-AF1^0^) and ERα-AF2 (ERα-AF2^0^) nuclear sub-functions or harboring the C451A mutation of the palmitoylation site that abrogates membrane ERα signaling (ERα-C451A), treated or not with E2 (**Figure 1A**). As previously demonstrated, B16K1 cells were selected as representative ER-negative tumors because their growth is increased by E2 *in vivo* similarly to LL2 (lung cancer) and 4T1 (breast cancer) cells, although they do not express ERα and do not proliferate *in vitro* in response to E2 10. Whereas B16K1 tumor growth was increased *in vivo* in ovariectomized (OVX) mice treated with E2 in all WT mice, the E2-induced tumor growth was completely lost in both the ERα-AF1^0^ and ERα-AF2^0^ mice (**Figure 1B-C**), demonstrating the crucial role of both the AF1 and AF2 transactivation functions of ERα for E2-induced tumor growth. Vessel density was then measured upon FITC-conjugated lectin injection in ERα-AF1^0^ mice treated or not with E2 (**Figure 1D**). No increase in vessel density was observed in ERα-AF1^0^ mice as compared to ERα-WT mice.

In the ERα-C451A mice which do not express the membrane isoform of ERα but retain its nuclear effects 22, the pro-tumoral effect of E2 on B16K1 tumor growth (**Figure 1E**) is also abrogated. The contribution of membrane effects of ERα was next evaluated using C57BL6/J mice treated with estrogen conjugated to a dendrimer (EDC), a selective activator of the extracellular signaling pathway of ERα 26 or its control dendrimer alone. In contrast to E2, EDC was able neither to promote tumor growth (**Figure 1F**), not to increase tumor angiogenesis as compared to E2 (**Figure 1G**).

Altogether, these results demonstrate that nuclear and membrane ERα signaling sensitizes the ERα-negative tumor growth while membrane ERα alone is not sufficient.

### Tamoxifen antagonizes the pro-tumoral effect of E2 on ERα-negative tumor growth by inhibiting the E2-induced tumoral angiogenesis

**Figure 2:**
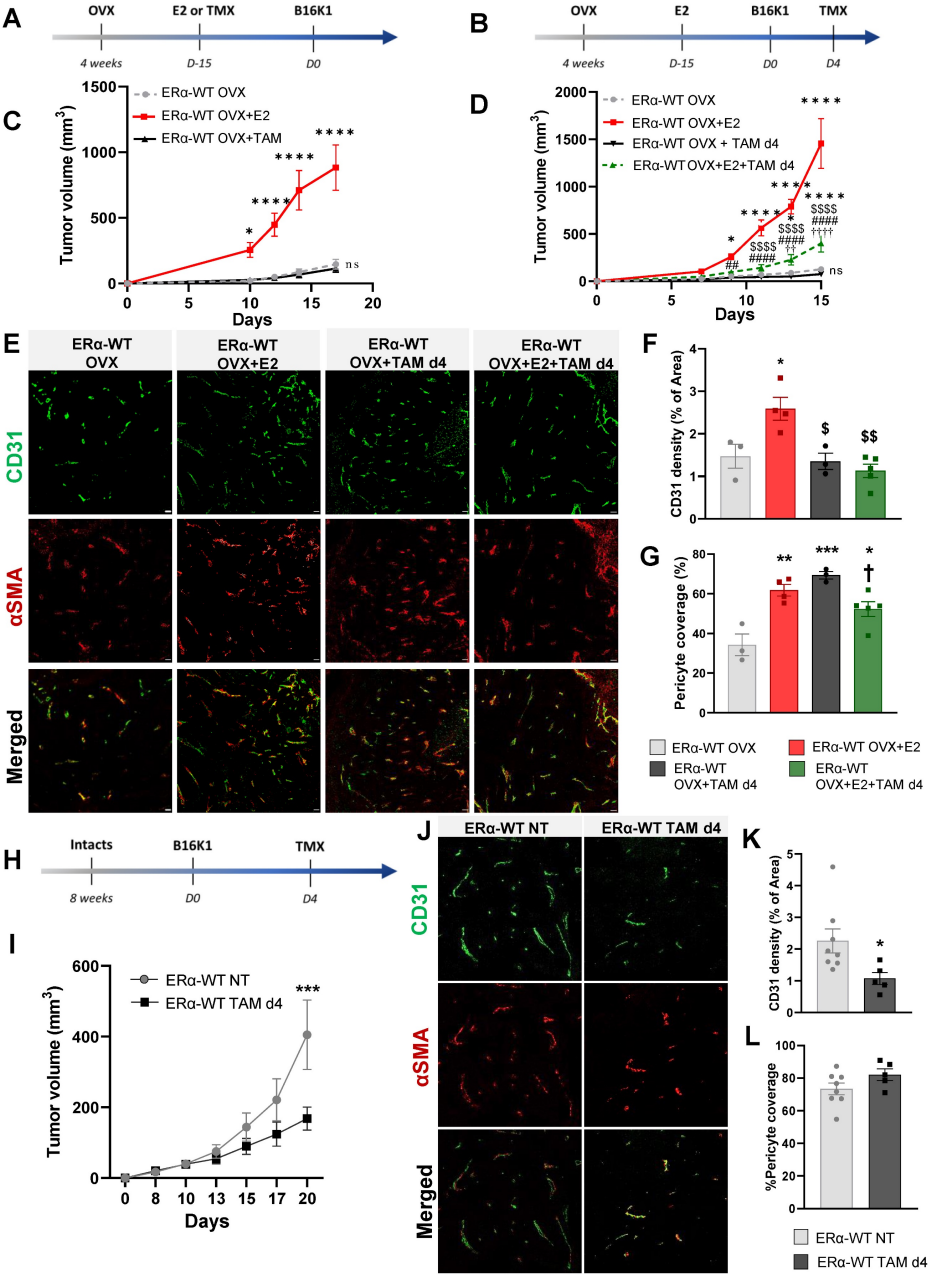
** Tamoxifen antagonized the pro-tumoral effect of E2 on ERα-negative tumors in both ovariectomized and intact mice by an inhibition of angiogenesis.** Tumors were established by subcutaneous injection of B16K1 cells in ovariectomized (OVX) C57BL/6 mice treated with E2 (E2) or Tamoxifen (TAM) alone or in combination with E2. (**A-D**) Tamoxifen treatment was initiated 15 days before tumor implantation such as E2 (A-C) or 4 days after tumor implantation (Day 4) (**B-D**). For (**C**) and (**D**), statistically relevant differences were estimated by two-way ANOVA followed by Bonferroni multiple comparison tests. n = 6-22 tumors/group. In **C**, * *P* < 0.05, **** *P* < 0.0001 presented differences between OVX+E2 and OVX+TAM or OVX; in **D** * *P* < 0.05, **** *P* < 0.0001 presented differences between OVX+E2, OVX+E2+TAM d4 and or OVX; ##* P* < 0.01, #### *P* < 0.0001 presented differences between OVX+TAM d4 and OVX+E2; $$ *P*< 0.01, $$$$ *P* < 0.0001 presented differences between OVX+E2+TAM d4 and OVX+E2 d4, †† *P* < 0.01, †††† *P* < 0.0001 presented differences between OVX+E2+TAM d4 and OVX+TAM d4. (**E**) Representative images of vessel density and pericyte coverage in B16K1 tumors harvested from ovariectomized (OVX) mice treated with E2 (OVX+E2) and Tamoxifen (TMX) alone or in combination (OVX+E2+TAM) (scale bars = 50µm). Quantification of (**F**) CD31 density, and (**G**) pericyte coverage. Significance was determined by one-way ANOVA followed by Bonferroni's multiple comparison tests, n = 3-5 tumors/group. Significant changes between control and treated groups (*** *P* < 0.001, **** P < 0.0001); between E2 alone and E2+TAM d4 ($ P< 0.05, $$ *P* < 0.01) or between TAM d4 alone and co-treatment E2+TAM d4 († *P* < 0.05). (**H-I**) Tumors were established by subcutaneous injection of B16K1 cells in intact C57BL/6 mice (ERα-WT) untreated (NT) or treated with Tamoxifen (TAM d4). Statistical relevant differences were estimated by two-way ANOVA followed by Bonferroni multiple comparison tests. N = 8-9 tumors/group. (**J**) Representative images of vessel density and pericyte coverage in B16K1 tumors harvested from intact mice untreated (NT) or treated with Tamoxifen (TAM) (scale bars = 50µm). (**K**) Quantification of CD31 density and (**L**) pericyte coverage. Significance was determined by Mann-Whitney U test, n = 5-8 tumors/group. Significant changes between control and treated groups (* *P* <0.05).

Tamoxifen, known as an ERα-AF1 partial agonist and an ERα-AF2 antagonist has largely demonstrated its efficacy to antagonize the proliferative effect of estrogen on ER-positive tumor proliferation 37. Here, we then tested the effects of tamoxifen on the model of B16K1 cells injected into WT immunocompetent mice. First of all, we compared to E2 the effect of tamoxifen alone when administered 15 days before tumor cell graft (**Figure 2A**) and we also investigated tamoxifen effects when tumors were already present by starting the treatment at day 4 after B16K1 cell injection in OVX mice (**Figure 2B**). Tamoxifen alone had no impact on tumor growth (**Figure 2C**) and upon an E2 treatment, tamoxifen antagonized the pro-tumoral effect of E2 on ERα-negative tumor growth (**Figure 2D**). Importantly, these data indicate that tamoxifen can efficiently reduce the growth of ERα-negative tumors.

Since we previously demonstrated that this E2-induced ER-negative tumor growth is dependent on the expression of ERα in endothelial cells, promoting a normalized angiogenesis and tumor growth 10, the effect of tamoxifen on vascular density and vessel normalization was evaluated using a double CD31 and pericyte marker α-smooth muscle actin (αSMA) immunostaining (representative pictures in **Figure 2E)**. Contrary to E2, which induced an increase of tumor vascular density 10, tamoxifen alone had no impact on vessel density (**Figure 2E-F**). Furthermore, when tamoxifen was injected on E2-treated mice, the vessel density was also decreased compared to the E2-treated mice, indicative of an inhibition of angiogenesis by tamoxifen treatment. Although tamoxifen treatment had a negative effect on tumor vascular density, pericyte coverage quantification indicates that tamoxifen leads to tumor vessel normalization in the same way than E2 treatment (**Figure 2E-G**).

The impact of tamoxifen was also evaluated in intact mice with endogenous circulating E2 (**Figure 2H).** Tamoxifen treatment initiated on day 4 after B16K1 cell injection also reduced growth of ERα-negative tumors in these intact mice (**Figure 2I).** Tamoxifen did also inhibit tumoral angiogenesis although it had no impact on pericyte coverage compared with untreated mice (**Figure 2J-L**).

Overall, these results demonstrate that tamoxifen reduced the growth of ERα-negative tumors through inhibition of angiogenesis but promotion of vessel normalization.

### Tamoxifen antagonizes the E2-induced angiogenesis *in vivo* and *ex vivo* in aortic ring assay

**Figure 3:**
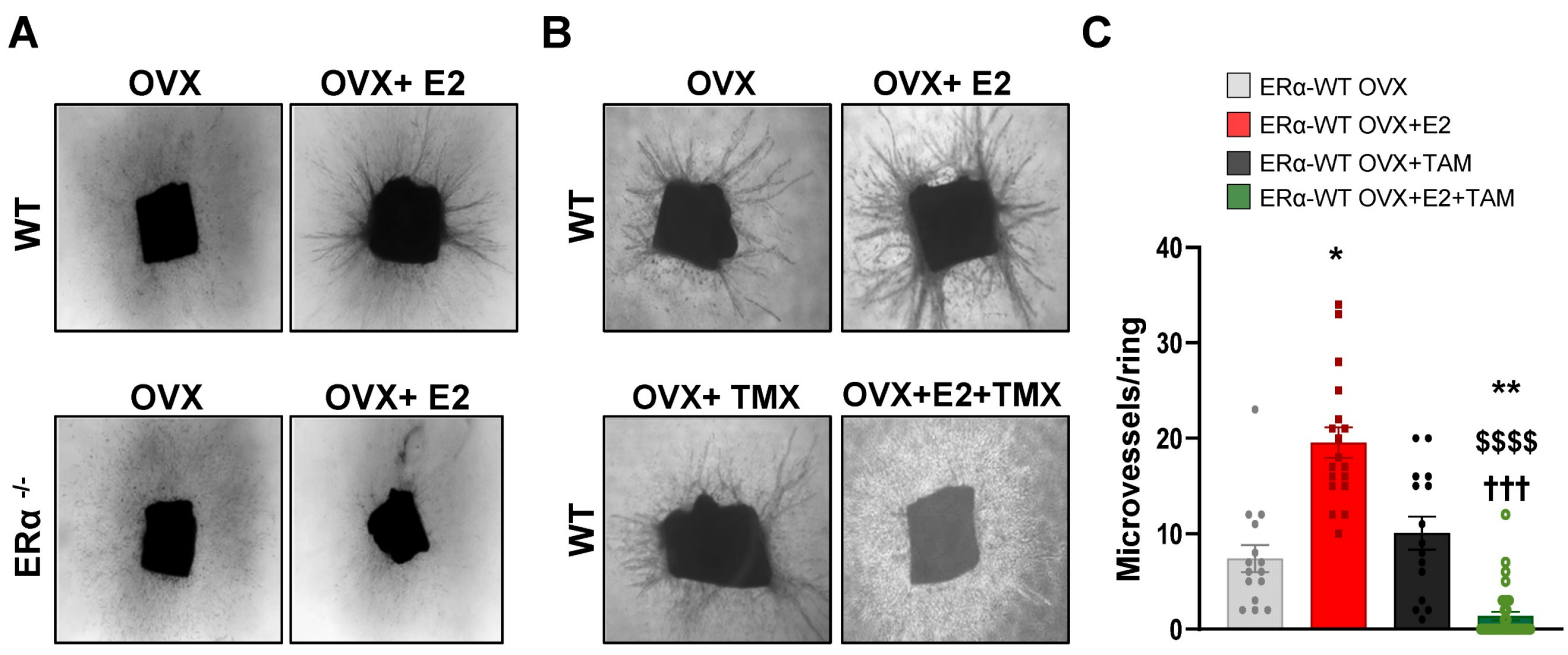
** E2-induced angiogenesis in aortic ring assay is ERα-dependent and antagonized by tamoxifen.** (**A**) Representative images (x40) of microvessel formations from aortic rings of ovariectomized WT or ERα-KO mice untreated (OVX) or treated with E2 for 2/3 weeks. n = 10-20 rings/condition. (**B**) Representative images (x40) of microvessel formation from aortic rings from ovariectomized WT mice untreated (OVX), treated with E2 (E2), Tamoxifen (TAM) or in combination (E2+TAM). (**C**) Quantifications of microvessel formations in each group are presented. Data presented are mean ± SEM. Statistical relevance was tested by Kruskal-Wallis test followed by Dunn's post-hoc test. Significant changes between control and treated groups (* P < 0.05, ** P < 0.01); between E2 alone and E2+4-OHT ($$$$ *P* < 0.0001) or between TMX and E2+TMX (**†††** *P* < 0.001).

We then analyzed vessel outgrowth in the *ex vivo* aortic ring model in ovariectomized mice treated or not with E2. These E2-induce effects on aortic vessel formation were fully dependent of ERα expression since these E2-induced effects were completely abrogated in aortic rings isolated from ERα-deficient mice treated or not with E2 (**Figure 3A**). To better explore the effect of tamoxifen on E2-dependent angiogenesis, we also analyzed vessel outgrowth in mice treated with tamoxifen, E2 or combined all together. Tamoxifen alone had no effect on the number of vessel outgrowths as already described 11. However, co-administration of tamoxifen with E2 drastically reduced the E2-induced vessel formation (**Figure 3B-C**).

Thus, these results confirm that tamoxifen inhibits the E2-induced angiogenesis outgrowth *ex vivo*.

### 4-hydroxytamoxifen elicits a differential transcriptional program from that of E2, while it fully antagonizes the E2 transcriptional effects in endothelial cells *in vitro*

**Figure 4:**
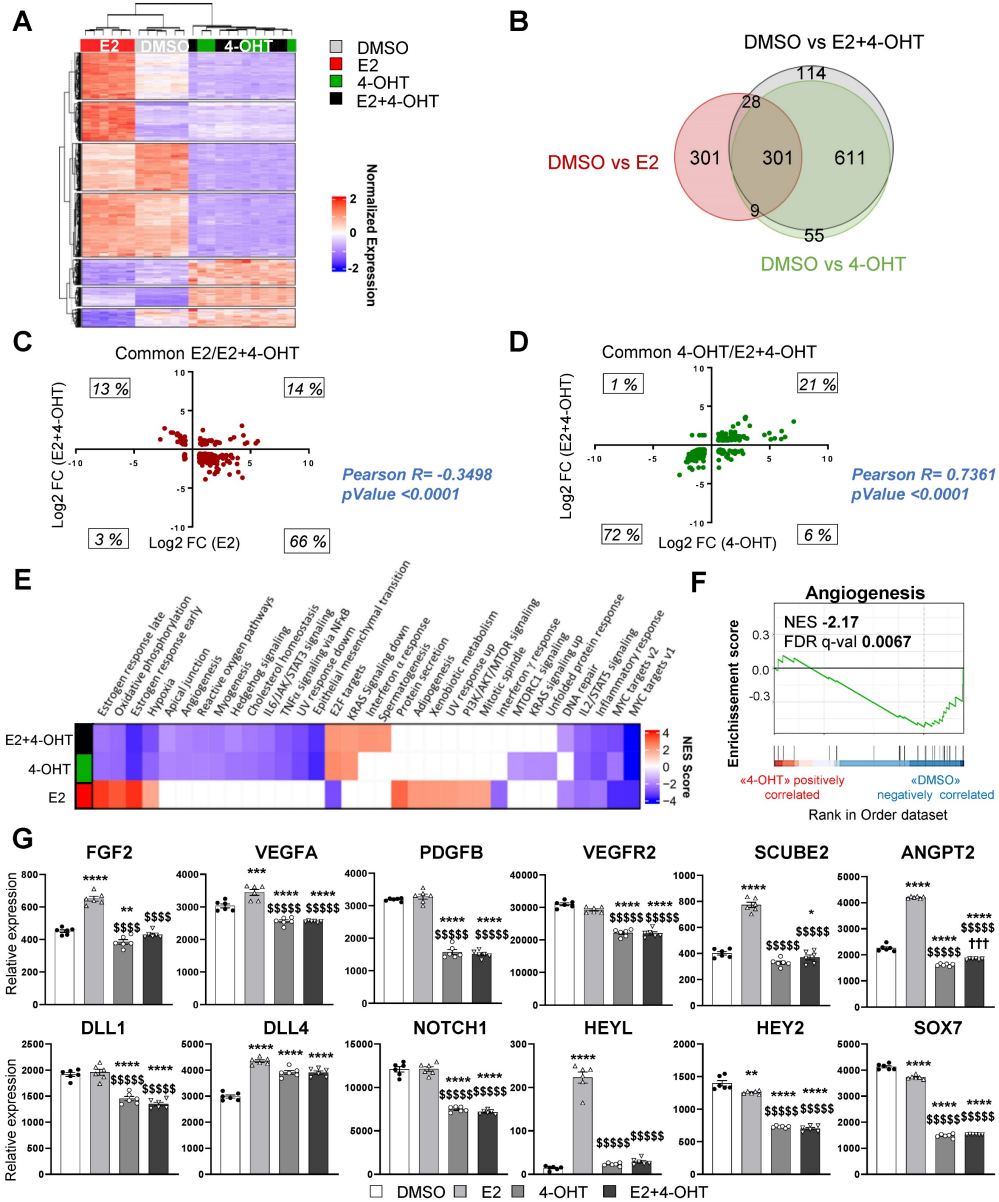
** 4-OHT induces its own transcriptional program in endothelial cells including an anti-angiogenic pathway.** mRNAs were isolated from TeloHAEC-endothelial cells expressing hERα-WT (n = 6 replicates per group) treated for 6 hours with E2 (1nM) or 4-OHT (100 nM) alone or in combination with E2. (**A**) The heatmap shown illustrates the relative normalized expression values of all genes significantly regulated following E2 or 4-OHT treatment alone or in combination as compared to vehicle (DMSO) (threshold for absolute fold change in expression (FC) >1.5 over DMSO control with a BH (Benjamini-Hochberg) corrected p-value < 0.05). HCL (Hierarchical Clustering) regroups each sample with its corresponding treatment group. (**B**) Venn diagram representing the overlap of differentially expressed genes between control and E2, 4-OHT or E2+ 4-OHT conditions. (**C**) Scatter plot assessing the correlation between the log2(FC) of genes commonly regulated in both E2 and E2+4-OHT conditions in the E2+4-OHT (Y axis) and E2 (X axis) conditions. (**D**) Similar analysis as in C on genes commonly regulated in E2 and E2+4-OHT conditions. (**E**) Normalized gene expressions were subjected to GSEA (Gene Set Enrichment Analysis) to identify functional pathways specific for E2, 4-OHT or E2+4-OHT treatments. The heatmap represents the NES (Normalized Enrichment Scores) values obtained on the significantly enriched MsigDb Functional Hallmarks (FDR q value < 0.05). NES are positive when the genes involved in a given pathway are up-regulated by the corresponding ligand *vs* the DMSO control, and negative in the converse scenario. (**F**) GSEA enrichment plot depicting the distribution of the enrichment scores of genes associated with the Angiogenesis MsigDb hallmark ranked along their expression change between OHT and DMSO conditions. (**G**) Normalized counts of TeloHAEC-endothelial cells expressing hERα-WT (n = 6 replicates per group) treated for 6 hours with E2 (1nM) or 4-OHT (100 nM) alone or in combination with E2. Data presented are as means ± SEM, while statistics calculated through a one-way ANOVA test followed by Bonferroni post-hoc test are illustrated as follows: significant changes between control and treated groups (* *P* < 0.05, ** P < 0.01, *** P < 0.001, **** P < 0.0001); significant differences between 4-OHT or E2 4-OHT and E2 conditions ($$ *P* < 0.01, $$$$ *P* < 0.0001).

To get insights into the molecular mechanisms of tamoxifen and E2 on endothelial cells, we performed RNA sequencing (RNA-Seq) on the ERα-transduced human endothelial cells (TeloHAEC-hERα-WT) that were acutely treated with E2, 4-hydroxytamoxifen (4-OHT, the active metabolite of tamoxifen) alone or in combination (E2+4-OHT). Gene regulations by E2, 4-OHT, vehicle and the co-treatment were compared and are presented on the heatmap (**Figure 4A**). The number of significantly regulated genes at a fold change >1.5 over vehicle with a BH adjusted *P*-value < 0.05 are presented in the Venn diagram (**Figure 4B**).

Importantly, the panels of genes regulated by E2 or 4-OHT over vehicle are substantially different. Indeed, E2 and 4-OHT differentially regulate 637 and 968 genes respectively, with only 301 commonly regulated genes. Interestingly, the co-treatment differentially regulates 1,044 genes but almost all of them are also found in the 4-OHT condition. This is consistent with the Principal Component Analysis (*PCA*) classification of the transcriptomes of the different samples (**Figure S1**) demonstrating that samples treated with 4-OHT alone or with the co-treatment are quite similar, as opposed to the ones treated with vehicle or E2.

More importantly, the analyses of the genes commonly regulated by E2 or by the co-treatment indicate that most of the genes are inversely regulated between these two treatments (**Figure 4C**). Indeed, most of the genes (66%) that are up-regulated by E2 are down-regulated by the co-treatment and inversely, 13 % of genes that are down-regulated by E2 are up-regulated by the co-treatment, with only 14 % and 3 % of genes being commonly regulated. In contrast, the variations of expression of the genes commonly regulated by 4-OHT and the co-treatment are going in the same direction (with 21% upregulated and 72 % down-regulated, **Figure 4D**). These results support that 4-OHT inhibits the transcriptional program initiated by E2 in endothelial cells and initiates a reprogramming of the transcription.

These observations correlate with the GSEA (Gene Set enrichment pathways) functional pathways analysis (**Figure 4E**). Indeed, treatments with E2 or 4-OHT alone substantially differ in terms of regulated pathways. Pathways such as early and late estrogen response are specifically upregulated by E2, while E2F targets (a group of genes that encodes a family of transcription factors) are pathways observed in the 4-OHT and the co-treatment conditions. Interestingly, these analyses identified the angiogenesis and the apical junction hallmarks as being down-regulated by 4-OHT in endothelial cells (**Figure 4F**, NES = -2,17 and FDR q-val = 0.0067).

Among the dysregulated genes, we found many genes encoding for angiogenic factors such as *VEGF-A, FGF-2, SCUBE2* and* ANGPT2,* or genes of the Notch signaling pathway such as *DLL4* and *HEYL* that are significantly upregulated by E2 and differentially regulated between E2 and 4-OHT (**Figure 4G**). In parallel, genes such as* VEGFR2, PDGFB, DLL1, NOTCH1, HEY2* and* SOX7* are significantly down-regulated by 4-OHT or the co-treatment. All these dysregulations were confirmed into 2 other independent experiments by RT-qPCR and are dependent of the ERα signaling since these effects are lost in non-transduced cells (**Figure S2**).

Altogether, these data demonstrate that tamoxifen antagonizes the E2-induced pathways, but also induces a specific transcriptional program in endothelial cells that inhibits angiogenesis.

### 4-hydroxytamoxifen differentially regulates glycolysis, oxidative phosphorylation and inflammatory response in endothelial cells

**Figure 5:**
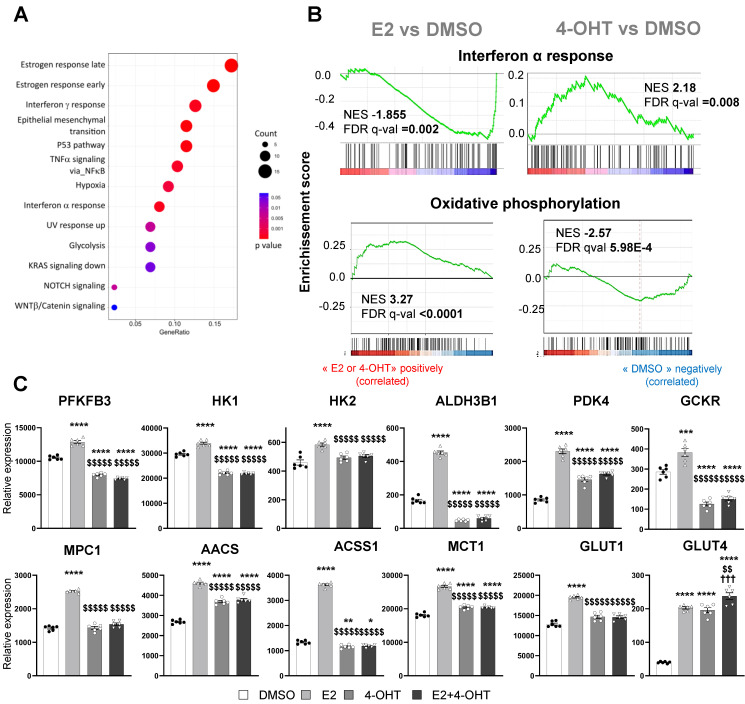
** E2 modulates EC inflammatory and glycolytic flux that is impeded by 4-OHT.** (**A**) Dot-plot illustrating the over-representation of MSigDb functional Hallmarks in the gene set comprising those whose regulations by E2 are antagonized by OHT. The sizes of the dots are proportional to the number of genes identified for each hallmark and their colors are scaled to the p-value. (**B**) GSEA enrichment plots depicting the distribution of the enrichment scores of genes associated with the Interferon α response and Oxidative phosphorylation MsigDb hallmark ranked along their expression change between E2 and DMSO (left side) or TAM and DMSO conditions (right side). (**C**) Normalized counts of TeloHAEC-endothelial cells expressing hERα-WT (n = 6 replicates per group) treated for 6 hours with E2 (1 nM) or 4-OHT (100 nM) alone or in combination with E2. Data presented are means ± SEM. Statistical relevance was measured by one-way Anova followed by Bonferroni post-test and is represented as follows: * P < 0.05, ** P < 0.01, ***P < 0.001, **** *P* < 0.0001 for the control *vs.* treated groups comparisons; and ($$ *P* < 0.01, $$$$ *P* < 0.0001) for the comparisons between the 4-OHT or E2 4-OHT *vs*. E2 conditions.

We then focused on the gene sets whose regulations by E2 are antagonized by 4-OHT (**Figure 5A**). Among these differentially regulated pathways, we found hallmarks for late and early estrogen response, the interferon α and γ response and also glycolysis. Gene sets associated with interferon α response were found to be enriched and positively regulated by 4-OHT while this hallmark was down-regulated by E2. Conversely, gene sets associated with oxidative phosphorylation were up-regulated by E2 and significantly down-regulated by 4-OHT (**Figure 5B**). More importantly, genes involved in glycolysis were highly differentially regulated between E2 and 4-OHT. Indeed, many glycolytic genes such as *PFKFB3, HK1, HK2, GCKR* and* ALDH3B1,* were significantly upregulated by E2 and not regulated at all or down-regulated by 4-OHT as compared to vehicle (**Figure 5C**). Expression of the *GLUT1* glucose transporter gene was upregulated by E2 and to a lesser extent by 4-OHT while expression of the less expressed *GLUT4* gene was upregulated by all treatments. Expression of some genes encoding for mitochondrial enzymes such as *PDK4*, *ACSS1* and *AACS* were also highly differentially dysregulated between E2 and 4-OHT (**Figure 5C**). Down-regulation of the pyruvate dehydrogenase kinase PDK4 is particularly relevant because this kinase is known as a gatekeeper directing the carbon flux into glycolysis and playing a central role in the switch from glycolysis to oxidative phosphorylation.

These data clearly show that, in endothelial cells, 4-OHT prevents the activation of pathways related to glycolysis, oxidative phosphorylation and inflammatory responses, inversely to E2.

### 4-hydroxytamoxifen differentially regulates transcription factors required to sustain E2 action in endothelial cells

**Figure 6:**
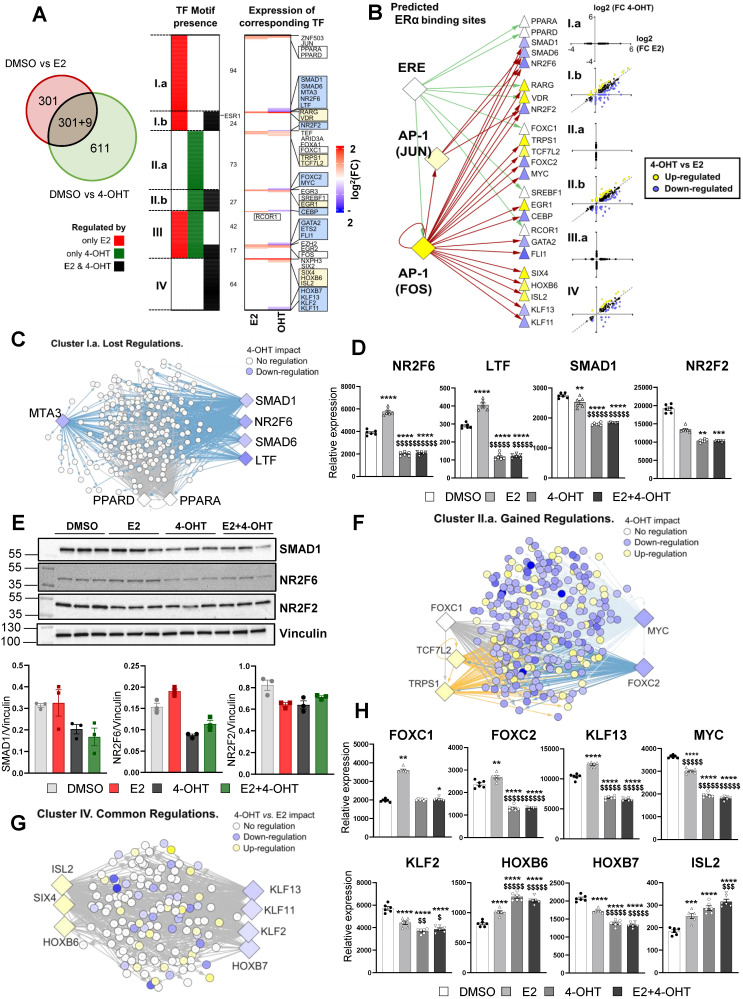
** 4-OHT may differentially regulate E2-sensitive genes by regulation of specific transcription factors expression.** (**A**) Enrichment for transcription factors (TF) motifs and binding sites determined in ENCODE studies was assessed within a -10/+10 kb window centered on the TSS of genes regulated either by E2, 4-OHT or both ligands. Some TF motifs were identified as being specific or common for genes present in the E2 and 4-OHT conditions, and classified within 7 clusters (from I.a including those only found around genes regulated by E2 only to cluster IV that integrates those identified only in genes regulated by either E2 and 4-OHT). The heatmap shown on the right side of the panel summarizes the variations of expression of the TFs binding to the identified motifs. Highlighted within the boxes are the names of the TFs whose expression are regulated differentially (blue or yellow boxes for down-or up-regulated genes in the 4-OHT condition vs. E2, respectively) or in the same manner (white boxes) by both ligands. In between the 2 heatmaps, the number of TFs belonging to each category is indicated. For instance, 94 motifs were specifically detected around genes regulated only by E2 (cluster I.a), whilst 42 were detected around genes regulated specifically by E2 or specifically by OHT (cluster III.a). (**B**) Summary scheme illustrating the presence of ERE or AP1 motifs allowing ERα mobilization around 10 kb from the TSS of the indicated genes encoding TF of interests defined from Panel A. The differential regulation of the expression of these TFs by E2 and 4-OHT is indicated as in the boxes from Panel A. (**C, F and G**) Cytoscape representation of networks linking the binding motifs of TF of interest (diamonds) to genes (circles) identified as carrying such motifs within the -10/+10 kb TSS window analyzed, respectively in the gene clusters Ia (lost regulations by OHT, Panel **C**), IIa (gained regulations, Panel** F**) and IV (common regulations, Panel **G**). The colors of the genes and TF nodes are representative of their fold-change in expression in the conditions indicated within the legends of each panel (4-OHT impact refers to the 4-OHT vs DMSO comparison). (**D and H**) Normalized counts in TeloHAEC-endothelial cells expressing hERα-WT (n = 6 replicates per group) treated for 6 hours with E2 (1nM) or 4-OHT (100 nM) alone or in combination with E2. Data presented are means ± SEM. Statistical relevance was measured by one-way ANOVA followed by Bonferroni post-test and is represented as follows: **** *P*<0.0001 for the control vs. treated groups comparisons; and $ *P*<0.05; $$ *P*<0.01 $$$$ *P*<0.0001 for the comparisons between the 4-OHT or E2 4-OHT vs. E2 conditions. (**E**) Western blot for SMAD1, NR2F2 and NR2F6 expression normalized to vinculin in TeloHAEC endothelial cells expressing hERα-WT treated for 6 hours with E2 (1 nM) or 4-OHT (100 nM) alone or in combination with E2 (n = 3 replicates per group).

To gain additional insights into these differential gene regulations between E2 and 4-OHT, we sought to identify specific transcription factors (TF) potentially involved in these processes. First, we focused on gene subsets specifically regulated by each treatment (only by E2 in red or only by 4-OHT in green, **Figure 6A**, left panel) or commonly by both treatments (black category). Then, we analyzed the proximal regions of these genes (defined as a 10 kb window centered on the transcription start site -TSS) for the presence of transcription factor (TF)-DNA binding elements (**Figure 6A**, left panel and **Table S2**). Interestingly, this analysis identified 7 clusters of TFs that are specific for a given subset of genes: 94 TFs for the genes regulated by E2 only (cluster I.a), 73 TFs for those regulated by 4-OHT only (II.b) and 64 TFs in the case of genes regulated by either E2 or 4-OHT (cluster IV). Some TFs were found to possibly bind near genes regulated by the 2 ligands (clusters III.a and III.b) or by E2 and both treatments (cluster I.b, 24 TFs) or by 4-OHT and both treatments (cluster II.b). Interestingly, ERα binding motifs such as estrogen response elements (EREs) were detected at the vicinity of genes regulated by E2 only or E2 and 4-OHT but not near those regulated only by 4-OHT (position highlighted within cluster I.b). To identify putative regulators that could explain 4-OHT adverse effects on E2-mediated regulations, we identified the transcriptional status of these TFs within our RNA-seq data (**Figure 6A**, right panel). We observed that a number of the identified TFs were differentially regulated by E2 and 4-OHT. The blue or yellow boxes highlight TFs that are down-regulated or up-regulated by 4-OHT, respectively. The white boxes depict TFs having lost their E2-mediated-regulation upon the action of 4-OHT. Strikingly, none of the identified TFs were down-regulated by E2 with a significant FC> 1.5. Importantly, genes encoding 24 of these 30 TFs were found to possess either an ERE in the vicinity of their TSS or JUN/FOS motifs on which ERα (*ESR1*) can be recruited through tethering the AP1 TF (**Figure 6B**).

Taken altogether, these analyzes nail some integrative regulations possibly involved in the distinct transcriptional activities of 4-OHT and E2 in the endothelial cells. For instance, among genes whose transcriptional regulations by E2 were not observed in 4-OHT conditions (Lost regulations, cluster I.a in **Figure 6A**), we found 4-OHT to down-regulate the expression of genes encoding *SMAD1, SMAD6, NR2F6* and* LTF* whilst being unable to recapitulate the up-regulation of *PPARA* and *PPARD* observed in the presence of E2 (**Figure 6C**). Binding motifs for these TFs were identified in 75 % of the genes belonging to this cluster I.a, grouped within the network shown within **Figure 6C.** We observed that 4-OHT decreases the expression of SMAD1, NR2F6 and LTF mRNAs and prevented their induction by E2 (**Figure 6D**), suggesting that, in combination, a slight reduction in the amounts or functionality of these TFs could at least in part explain why these genes are not regulated by 4-OHT.

Similarly, 81.5% of the genes belonging to the cluster I.b, which also comprises genes that are not regulated by 4-OHT but for which the co-treatment with 4-OHT affects E2-regulations, could be linked to 4-OHT down-regulation of *NR2F2* (also known as COUPTFII, an important factor in endothelial cell growth, **Figure 6D**) and upregulation of *RARG* and *VDR* (not shown). We confirmed that these TFs were regulated on a ERα-dependent manner by RT-qPCR (**Figure S3**) and also confirmed regulations of NR2F6, SMAD1 and NR2F2 proteins by western blot using specific antibodies (**Figure 6E**). Interestingly, we found that the 4-OHT mediated up-regulation of *TRPS1* and *TCF7L2* together with the down-regulation of *MYC* and *FOXC2* or loss of regulation of *FOXC1* are associated with 271 (48%) of the genes regulated only by 4-OHT (cluster II.a, cf **Figure 6F-H**) which are mostly down-regulated genes (**Figure 6B**). Finally, within the TFs whose motifs were identified around genes regulated by both E2 and 4-OHT (cluster IV) the down-regulations of *KLF13/KLF11* and coincident up-regulations of *HOXB6/SIX4* and *ISL2* by 4-OHT could be linked to 100% of the genes whose regulations are statistically different between both ligands (**Figure 6G-H**).

Altogether, these data suggest that E2 and 4-OHT regulations involve specific transcription factors and that 4-OHT is likely to impair the regulation of many E2-sensitive genes by downregulating the expression of specific transcription factors such as SMAD1, SMAD6, MTA3, NR2F6, LTF, NR2F2, ETS2, FLI1, KLF2, KLF1 and KLF13.

## Discussion

In the present study, the respective role of membrane and nuclear ERα signaling of E2 on ER-negative tumor growth and angiogenesis was evaluated in parallel with the effects of tamoxifen, which was the first clinically approved ER-targeted agent. Using transgenic mice mutated on ERα subfunctions, we uncovered that both nuclear and membrane ERα signaling are necessary for E2-induced ERα-negative tumor growth while activation of membrane ERα alone by EDC was not sufficient. This is consistent with the absence of ERα-negative tumor growth acceleration by E2 in ERα-AF2^0^ mice where there is a specific loss of nuclear ERα signaling with preservation of membrane ERα signaling 22, 38. These findings then indicate that ERα-negative tumor growth and angiogenesis requires both nuclear and membrane actions of ERα, that is one of the first vascular effects of E2 that implicates a contribution of these two effects 39.

We also explored the actions of tamoxifen in ERα-negative tumors growth and angiogenesis, since we recently demonstrated the complexity of actions of tamoxifen on vascular effects. Indeed, using mice harboring mutated specific functions of ERα, previous work of our laboratory evidenced that tamoxifen acts *in vivo* through nuclear ERα activation, and more precisely through the AF1 to accelerate endothelial healing in the endovascular model of carotid injury due to a direct action of smooth muscle cells 40 or to prevent neo-intimal hyperplasia 41. At the same time, it blocks the direct action of E2 on the endothelium and thereby inhibits reendothelialization in a model where the underlying smooth muscle cells are absent 40, 42, demonstrating that tamoxifen directly antagonizes the actions of E2 membrane activation of ERα 22, 39. In this study, we found that tamoxifen alone has no impact on tumor growth of ERα-negative tumor in ovariectomized mice. However, tamoxifen antagonizes the stromal membrane and nuclear actions of ERα and thus prevents the E2 pro-tumoral effects, due to an inhibition of angiogenesis observed both *in vivo,* and *ex vivo,* in aortic ring assays. The tamoxifen-induced inhibition of angiogenesis probably involves its antagonist actions on AF2 on endothelial cells, that are well documented in cultured cancer cells. It also inhibits membrane actions of E2 that have been implicated *in vitro* in endothelial cell migration, in which the E2-stimulated PI-3-kinase activity was blocked by tamoxifen 43. Moreover, in the tumoral context, the interactions between endothelial cells and smooth muscle cells are important for vessel normalization. In tumors, tamoxifen reduces angiogenesis but still increases pericyte coverage, that might have functional consequences in drug delivery 44. This can be compared to tamoxifen acceleration of re-endothelialization following mechanical injury where tamoxifen was found to impact smooth muscle cells, demonstrating that tamoxifen affects differently endothelial cells and pericytes as compared to E2 40. Indeed, many studies reported a dissociation between increased angiogenesis and normalization of the vessels. On the one hand, the SHP2 tyrosine phosphatase or the neural adhesion molecule L1 deletion reduced tumor angiogenesis but promoted vascular normalization 45, 46. On the other hand, Pten deletion enhanced angiogenesis and caused an impaired recruitment of pericytes 42.

This study supports data of clinical trials showing the efficacy of tamoxifen not only on ERα-positive breast tumors where it acts by competing with E2 for binding to the ER in the tumor tissue, but also in breast tumors without ER where 5-10% response rate has been reported 3, 4. Moreover, in clinical trials of patients with melanoma treated with chemotherapy including tamoxifen or not, the inclusion of tamoxifen improved the overall and the partial response 47. In experimental murine models, tamoxifen has also shown some efficacy in reducing tumor volume and blood vessels network in lung tumors of female mice when compared to control mice 12.

Interestingly, our large-scale transcriptomic analyses on endothelial cells revealed that 4-OHT, a metabolite of tamoxifen, not only antagonizes transcription of some E2-induced genes, but also elicits its own transcriptional response. Transcriptional analysis of the impact of 4-OHT has been widely performed in MCF7 or in tamoxifen-resistant breast cancers 48, 49 since tamoxifen remains an important drug in the adjuvant and metastasis setting of ERα-positive tumors. This is the first time to our knowledge that this transcriptional effect of tamoxifen was evaluated in endothelial cells. In contrast to breast cancer MCF7 cells 48, in which 13% of the genes induced by E2 are also upregulated by tamoxifen and only 3% of E2- induced genes are down-regulated by tamoxifen, a majority of the upregulated genes by E2 (66%) were down-regulated by 4-OHT in endothelial cells. These data demonstrate that the transcriptional response of tamoxifen in breast cancer cells differs from that of endothelial cells 14, 50, 51.

More importantly, we also demonstrate that 4-OHT-tamoxifen differentially regulates not only the pro-angiogenic growth factors in endothelial cells, but also modulates glycolysis. This is particularly relevant in the therapeutic prospective of cancer treatment since a new paradigm for modulating angiogenesis has emerged by targeting endothelial metabolism and not the angiogenic factors that have demonstrated some limits in the success of VEGF-targeted therapy. Indeed, many studies have recently highlighted how endothelial cells adapt their metabolism to proliferate or migrate during vessel sprouting with metabolism co-determining vessel sprouting 52, 53. Hyperproliferative endothelial cells in cancer have high rates of glycolysis to optimize their energy production. One subject of intense research so far was the study of the role of the 6-phosphofructose-2-kinase fructiose-2,6-biphosphatase 3 (PFKFB3), that is generally upregulated in tumor endothelial cells 54. This gene is differentially regulated by E2 and tamoxifen, together with many genes of this glycolytic pathway including the hexokinase 1, 2 and 4 (*HK1, HK2, GCKR* also known as hexokinase 4) and the Aldehyde Dehydrogenase 3 Family Member B1 (*ALDH3B1*). Meanwhile, the oxidative phosphorylation pathway is also upregulated in E2 conditions as compared to 4-OHT or the co-treatment to sustain biosynthetic processes, such as aspartate production during proliferation with E2-induced upregulation of *AACS, ACSS1,* and* PDK4* that are partially or totally inhibited by tamoxifen.

Finally, transcription factor binding sites analyses on the genome regions surrounding the genes specifically regulated by E2 or 4-OHT, revealed that the regulation of E2 and 4-OHT responsive genes in endothelial cells presumably requires different sets of transcription factors. Moreover, 4-OHT was found to particularly down-regulate transcription of genes encoding some of these specific factors, such as NR2F2 (also known as COUP-TFII) and NR2F6 (EAR2, or V-erbA-related gene), two specific transcription factors involved in endothelial functions. Interestingly, COUP-TFII (NR2F2) is a transcription factor highly expressed in endothelial cells, which regulates Notch signaling pathways 49 and is also involved in endothelial identity, since the choice of arterial versus venous fate is a result of a cross-talk between Notch and COUP-TFII signaling 55. Interestingly, NR2F6 (EAR2) was also shown to interact with COUP-TFII signaling 56, which probably explains why their motifs were found together at the vicinity of similar gene subsets (see **Figure 6B**). Some of these key transcription factors, such as ETS2 involved in the survival of endothelial cells and regulation of the angiogenic processes 57, SMAD1 known to modulate the angiogenic program and NR2F6 (EAR2) are specifically regulated by 4-OHT as compared to E2. These data reinforce the dynamics of ER recruitment of transcription factors in endothelial cells depending on the ligands, such as that was observed for breast cancer cell lines 48.

This present work is also relevant to most widely used strategies for temporal control of genetic manipulation that commonly inject tamoxifen in Cre-ER^T2^-LOXP transgenic mouse models for lineage tracing studies and particularly for studies in vascular pathologies. Moreover, in this context, the experimental doses of tamoxifen for mice are often 100 times higher than those used in human breast cancer therapy. Tamoxifen injection might impair angiogenesis in these mouse models independently of the deleted floxed gene and might therefore impair the interpretation of the data. For instance, tamoxifen injection impairs retinal angiogenesis independently of gene deletion, requiring to include not only tamoxifen-injected Cre-ER negative littermates but also tamoxifen-injected cre-ER control mice lacking flox genes 58.

To conclude, this present work demonstrates that: 1) both nuclear and membrane stromal ERα modulate ER-negative tumor growth and that these effects of E2 are antagonized by tamoxifen; 2) tamoxifen blocks angiogenesis by altering the angiogenic growth factors, and endothelial metabolism, using a specific transcriptional program and key transcription factors. As a consequence of the importance of angiogenesis and vascularization of tumors for the other treatments, the data provided by this study have to be integrated in therapeutic strategies (radio, chemo- and immunotherapies) that all are influenced by the tumor microenvironment.

## Supplementary Material

Supplementary figures and tables.
