# Supplementary material for: Reprogramming of endothelial gene expression by tamoxifen inhibits angiogenesis and ERα-negative tumor growth
Source: Theranostics. 2024 Jan 1;14(1):249–64. doi: 10.7150/thno.87306 (PMC10750193; doi:10.7150/thno.87306)
Supplement: Supplementary file 1 — Supplementary figures and tables. [file thnov14p0249s1.pdf]

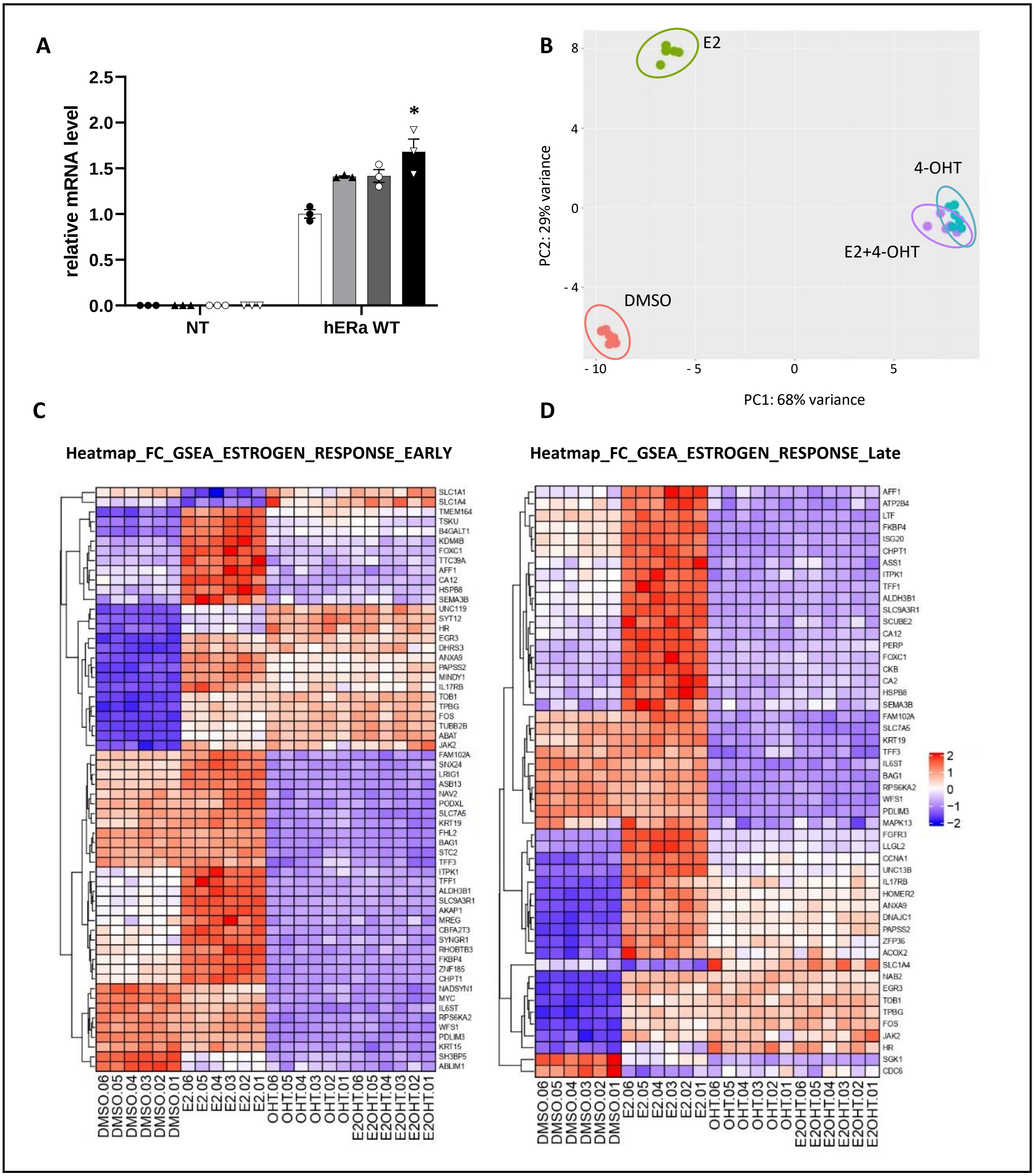

Figure S1: mRNAs were isolated from ERα-transduced endothelial cells treated by E2, TAM or the combination of E2+TAM compared to vehicle (DMSO) and sequenced. **(A)** Relative expression of ESR1 mRNA in TeloHAEC cells transduced with the human ESR1 gene following the different treatments**, (B)** PCA demonstrated the source of variance in our data following the different treatments, showing that Tam and E2+Tam are quite similar as opposed to E2 and vehicle. **(C-D)** Heat-map representative of the differentiated genes in the Estrogen Early response and Estrogen late response pathway identified from GSEA. Statistical relevance was measured by Kruskal-Wallis followed by Dunn's post test and is represented as follows: **P*<0.05, for the control *vs.* treated groups comparisons in hERα-WT cell line.

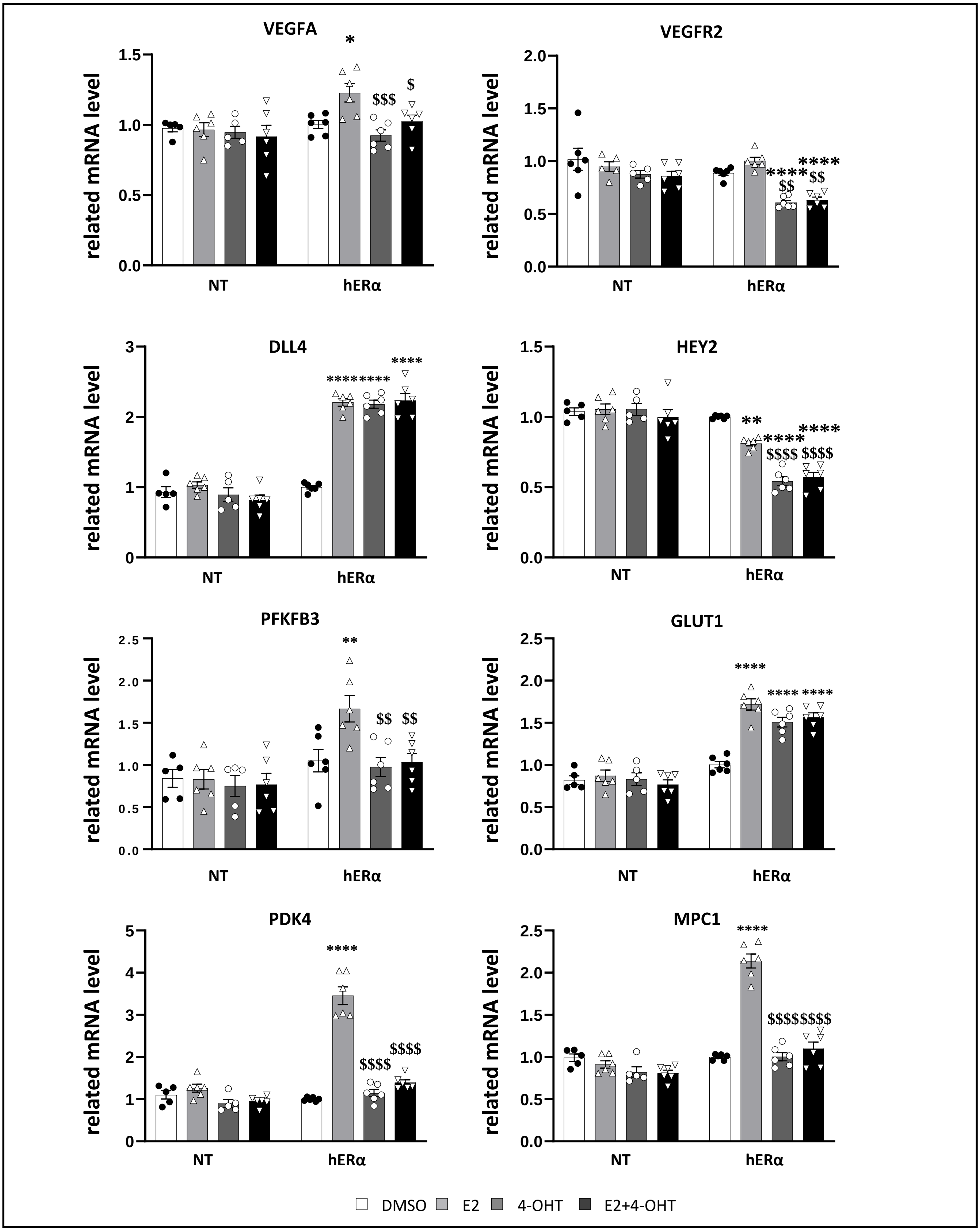

Figure S2: Relative expression of angiogenic and cell metabolism mRNAs in TeloHAEC cells transduced or not with the human ESR1 gene following the different treatments (n = 5-6 per group from 2 independent experiments). Statistical relevance was measured by one-way ANOVA followed by Bonferroni post test and is represented as follows: * *P*<0.05, ** *P*<0.01, ****P*<0.001, *****P*<0.0001 for the control *vs*. treated groups comparisons; and ($$ *P*<0.01, $$$$ *P*<0.0001) for the comparisons between the 4-OHT or E2+4-OHT *vs*. E2 conditions.

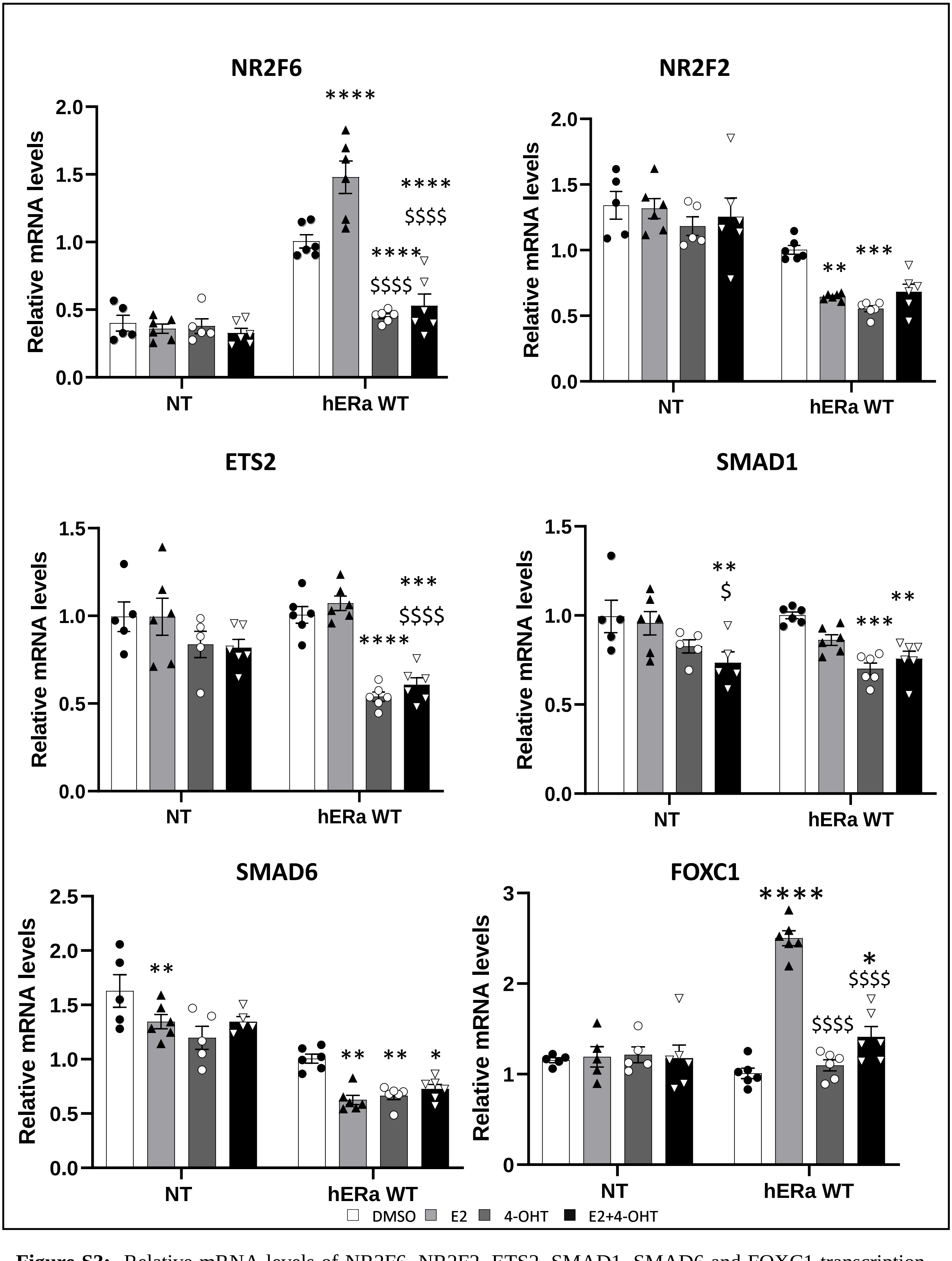

Figure S3: Relative mRNA levels of NR2F6, NR2F2, ETS2, SMAD1, SMAD6 and FOXC1 transcription factors in TeloHAEC transduced or not with the human ESR1 gene following the different treatments (n =6 per group from 2 independent experiments). Statistical relevance was measured by two-way ANOVA followed by Bonferroni's post test. : * $P<0.05$, ** $P<0.01$, ***$P<0.001$, ****$P<0.0001$ for the control *vs.* treated group comparisons; and ($$ $P<0.01$, $$$$ $P<0.0001$) for the comparisons between the 4-OHT or E2+ 4-OHT *vs*. E2 conditions.

Gene symbol	NCBI Reference Sequence	Species	Forward Sequence (5'-3')	Reverse Sequence (5'-3')
ESR1	NM_000125	*Homo Sapiens*	GAGTCTGGTCCTGTGAGGGCT	TGGTTGGTGGCTGGACACATA
VEGFA	NM_001171623	*Homo Sapiens*	TGCTGTCTTGGGTGCATTGGA	CCACTTCGTGATGATTCTGCC
HEY2	NM_012259	*Homo Sapiens*	ACGACCTCCGAGAGCGACAT	ATCCGATCCCGACGCCTTTTC
DLL4	NM_019074	*Homo Sapiens*	CTGTGCCAACGGGGGACAGTG	GTGGGCGCAAGGGTTACGGG
PFKFB3	NM_004566	*Homo Sapiens*	CCGAGTGCAGAAGATCTGGGT	TGGGGGAGTTGGTCAGCTTTG
GLUT1	NM_006516	*Homo Sapiens*	CCCTGCAGTTTGGCTACAACA	ATAGCGGTGGACCCATGTCTG
PDK4	NM_002612	*Homo Sapiens*	GCCTGTGAGACTCGCCAACAT	GCTTTCTGGTCATCTGGGCTTTT
MPC1	NM_016098	*Homo Sapiens*	CATGAGTACGCACTTCTGGGGC	AGTTCCGAGGCTGTACCTTGT
HPRT	NM_000194	*Homo Sapiens*	TGCTTTCCTTGGTCAGGCAGT	CTTCGTGGGGTCCTTTTCACC
NR2F6	NM_005234	*Homo Sapiens*	CGGCAAGCATTACGGTGTCTT	ATCTGGCAGTCACGGTTGGAC
NR2F2	NM_021005	*Homo Sapiens*	AGGCCATAGTCCTGTTCACCTC	AAAGCTTTCCGAATCTCGTCGG
ETS2	NM_005239	*Homo Sapiens*	GCAGCGGCAGGATGAATGA	CCAAGCCTGTTGGCACTTCTT
SMAD1	NM_005900	*Homo Sapiens*	TCCCCTGCCCTCAGAAATCAA	CATGGAACGCTTCACCCACAC
SMAD6	NM_005585	*Homo Sapiens*	ACCTCCCTACTCTCGGCTGT	AGACATGCTGGCGTCTGAGAA
FOXC1	NM_001453	*Homo Sapiens*	TTCGAGTCACAGAGGATCGGC	CGGTACAGAGACTGGCTGGAA
VEGFR2	NM_002253	*Homo Sapiens*	CCCAGATGACAACCAGACGGA	GCCACAGACTCCCTGCTTTTG

<u>Table S1 :</u> : List of primers for RT-qPCR.

Unprocessed western blots presented on Figure 6E

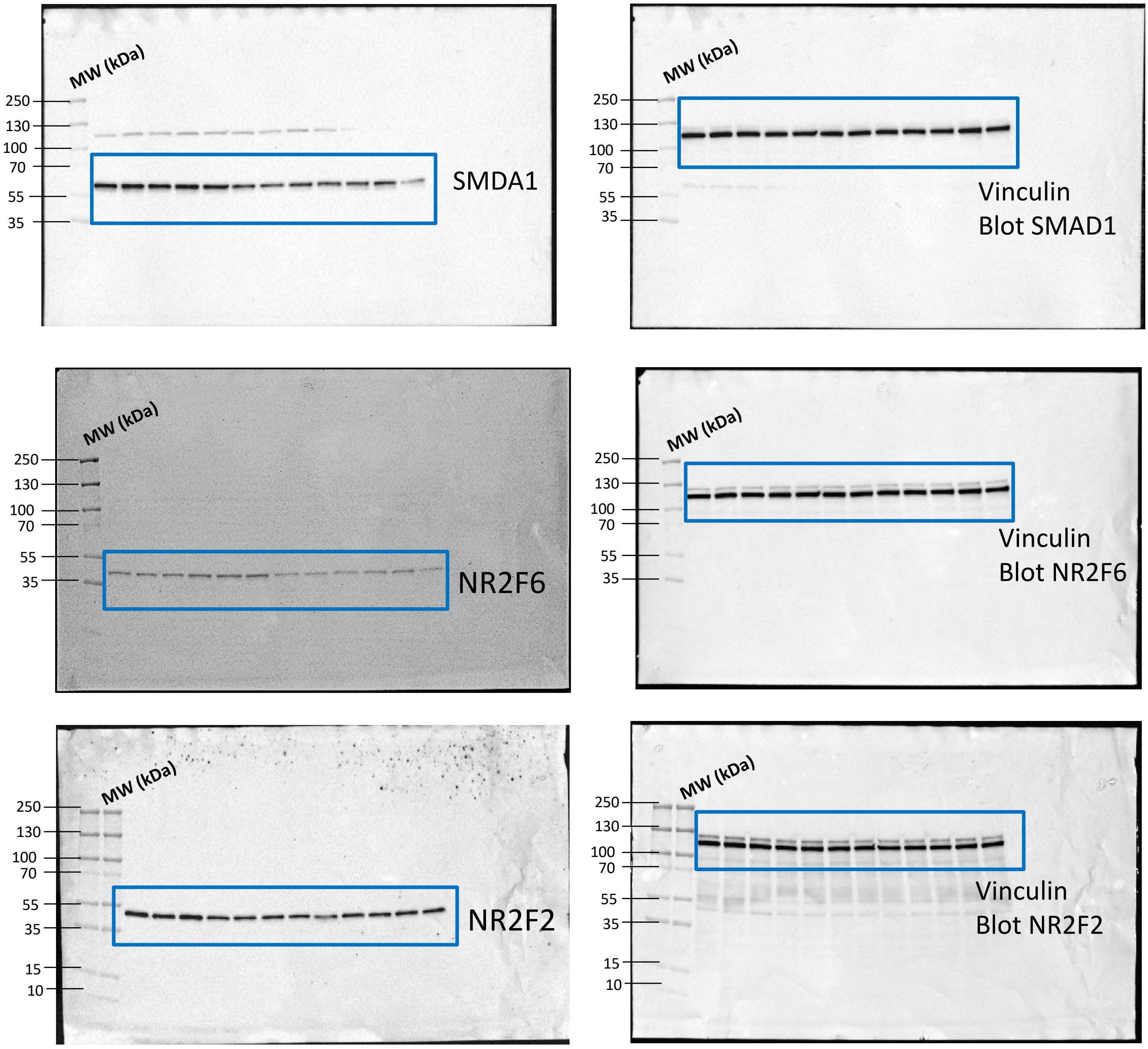

Table S2: Matrice TFs expression motifs			
**Category**	**TF**	**FC E2**	**FC OHT**
All	EGR2	1.44301760835644	2.43652325515723
All	EZH2	0.743958085909748	0.873715941217246
All	FOS	0.964715101776263	1.05330035011308
All	ZBTB7A	0.638236214989486	0.887048140986128
All	POLR2A	0.652989162329787	0
All	ATF3	0	0
All	CTCF	0	0
All	EP300	0	0
All	ESRRA	0	0
All	HDAC2	0	0
All	IRF1	0	0
All	MAZ	0	0
All	MXI1	0	0
All	NR2C2	0	0
All	RAD21	0	0
All	SP1	0	0
All	WT1	0	0
E2_only	ZNF503	1.14832308897238	1.90477969349746
E2_only	FOSB	1.0247636900742	1.01316623642807
E2_only	JUNB	0.992384839139608	0.965347662654313
E2_only	PPARA	0.98215458456039	0
E2_only	PPARD	0.620800809642421	0
E2_only	AKR1A1	0	0
E2_only	ASCL1	0	0
E2_only	BACH2	0	0
E2_only	BATF	0	0
E2_only	CTCFL	0	0
E2_only	EBF1	0	0
E2_only	EVX1	0	0
E2_only	EVX2	0	0
E2_only	FOSL1	0	0
E2_only	FOSL2	0	0
E2_only	GRHPR	0	0
E2_only	GTF3C2	0	0
E2_only	HAND1	0	0
E2_only	HLCS	0	0
E2_only	HNF4A	0	0
E2_only	HNF4G	0	0
E2_only	HSF4	0	0
E2_only	HSPA1L	0	0
E2_only	IKZF1	0	0
E2_only	IRF2	0	0
E2_only	IRF3	0	0
E2_only	IRF4	0	0
E2_only	IRF5	0	0
E2_only	IRF6	0	0
E2_only	IRF7	0	0

E2_only	IRF8	0	0
E2_only	IRF9	0	0
E2_only	JDP2	0	0
E2_only	KDM5A	0	0
E2_only	MTHFD1	0	0
E2_only	NANOG	0	0
E2_only	NFIA	0	0
E2_only	NFIB	0	0
E2_only	NFIX	0	0
E2_only	NFKB1	0	0
E2_only	NR1D1	0	0
E2_only	NR1H2	0	0
E2_only	NR1H3	0	0
E2_only	NR1H4	0	0
E2_only	NR1I2	0	0
E2_only	NR1I3	0	0
E2_only	NR2E3	0	0
E2_only	NR4A1	0	0
E2_only	NR4A2	0	0
E2_only	NR4A3	0	0
E2_only	NR5A1	0	0
E2_only	NR5A2	0	0
E2_only	NUP133	0	0
E2_only	PIR	0	0
E2_only	PPARG	0	0
E2_only	PRDM1	0	0
E2_only	RARA	0	0
E2_only	RBM17	0	0
E2_only	RNF114	0	0
E2_only	RORA	0	0
E2_only	RXRA	0	0
E2_only	RXRB	0	0
E2_only	RXRG	0	0
E2_only	SMAD2	0	0
E2_only	SMAD3	0	0
E2_only	SMAD4	0	0
E2_only	SMAD5	0	0
E2_only	SMAD9	0	0
E2_only	SMAP2	0	0
E2_only	SMARCC1	0	0
E2_only	SMC3	0	0
E2_only	SMPX	0	0
E2_only	STAT1	0	0
E2_only	STAT2	0	0
E2_only	STAT3	0	0
E2_only	TCEAL6	0	0
E2_only	THRA	0	0
E2_only	THRB	0	0
E2_only	TLX1	0	0
E2_only	TRIM21	0	0

E2_only	TRMO	0	0
E2_only	UBP1	0	0
E2_only	ZCCHC17	0	0
E2_only	ZFP2	0	0
E2_only	ZNF436	0	0
E2_only	ZNF571	0	0
E2_only	ZNF595	0	0
E2_only	ZNF671	0	0
E2_only	ZNF71	0	0
E2_only	ZNF766	0	0
E2_only	SMAD1	0	-0.609981710518522
E2_only	SMAD6	0	-0.653329633302331
E2_only	MTA3	0	-0.862969779741773
E2_only	NR2F6	0	-0.953539304081849
E2_only	LTF	0	-1.27903625168257
OHT_only	TEF	1.11130304856721	0.62440889886011
OHT_only	ARID3A	0.864167319107167	0.673512686947838
OHT_only	FOXA1	0.883081087386496	0.668667643031372
OHT_only	FOXC1	0.874904220927108	0
OHT_only	TRPS1	0	0.738753974455545
OHT_only	TCF7L2	0	0.659821888662243
OHT_only	ATF2	0	0
OHT_only	BCLAF1	0	0
OHT_only	BHLHE40	0	0
OHT_only	CCNT2	0	0
OHT_only	CREB1	0	0
OHT_only	CUX1	0	0
OHT_only	DBP	0	0
OHT_only	E2F1	0	0
OHT_only	E2F6	0	0
OHT_only	FOXA2	0	0
OHT_only	FOXA3	0	0
OHT_only	FOXB1	0	0
OHT_only	FOXD1	0	0
OHT_only	FOXD2	0	0
OHT_only	FOXD3	0	0
OHT_only	FOXD4	0	0
OHT_only	FOXD4L1	0	0
OHT_only	FOXD4L3	0	0
OHT_only	FOXD4L4	0	0
OHT_only	FOXD4L5	0	0
OHT_only	FOXD4L6	0	0
OHT_only	FOXF2	0	0
OHT_only	FOXL2	0	0
OHT_only	FOXM1	0	0
OHT_only	GATA1	0	0
OHT_only	GATA3	0	0
OHT_only	GATA4	0	0
OHT_only	GATA5	0	0
OHT_only	GATA6	0	0

OHT_only	GCM1	0	0
OHT_only	HCFC1	0	0
OHT_only	HIC1	0	0
OHT_only	HLF	0	0
OHT_only	KAT2A	0	0
OHT_only	MAX	0	0
OHT_only	MBD4	0	0
OHT_only	NFE2	0	0
OHT_only	NFIL3	0	0
OHT_only	NHLH1	0	0
OHT_only	PBX3	0	0
OHT_only	PHF8	0	0
OHT_only	REST	0	0
OHT_only	RUNX1	0	0
OHT_only	SIN3A	0	0
OHT_only	SOX8	0	0
OHT_only	TAF1	0	0
OHT_only	TBX15	0	0
OHT_only	TFAP2B	0	0
OHT_only	TRIM28	0	0
OHT_only	USF1	0	0
OHT_only	ZC3H11A	0	0
OHT_only	ZEB1	0	0
OHT_only	ZMIZ1	0	0
OHT_only	ZNF143	0	0
OHT_only	ZNF16	0	0
OHT_only	ZNF189	0	0
OHT_only	ZNF215	0	0
OHT_only	ZNF23	0	0
OHT_only	ZNF263	0	0
OHT_only	ZNF274	0	0
OHT_only	ZNF429	0	0
OHT_only	ZNF471	0	0
OHT_only	ZNF502	0	0
OHT_only	ZNF607	0	0
OHT_only	ZNF84	0	0
OHT_only	FOXC2	0	-0.854467281393582
OHT_only	MYC	0	-0.896244922724527
Common_E2_OHT	NXPH3	2.1675180632467	2.77857180621833
Common_E2_OHT	SIX2	1.31871763429417	1.61248549093675
Common_E2_OHT	SIX4	0	0.7869628524257
Common_E2_OHT	HOXB6	0	0.63057364100111
Common_E2_OHT	ISL2	0	0.64678158173597
Common_E2_OHT	ATF6	0	0
Common_E2_OHT	ATF6B	0	0
Common_E2_OHT	BSX	0	0
Common_E2_OHT	CDX1	0	0
Common_E2_OHT	CDX2	0	0
Common_E2_OHT	CDX4	0	0
Common_E2_OHT	CHURC1	0	0

Common_E2_OHT	CPSF4	0	0
Common_E2_OHT	CREB3	0	0
Common_E2_OHT	GPD1	0	0
Common_E2_OHT	HOXA11	0	0
Common_E2_OHT	HOXA13	0	0
Common_E2_OHT	HOXA6	0	0
Common_E2_OHT	HOXA7	0	0
Common_E2_OHT	HOXB13	0	0
Common_E2_OHT	HOXC11	0	0
Common_E2_OHT	HOXC12	0	0
Common_E2_OHT	HOXC13	0	0
Common_E2_OHT	HOXC6	0	0
Common_E2_OHT	HOXD1	0	0
Common_E2_OHT	HOXD13	0	0
Common_E2_OHT	KLF1	0	0
Common_E2_OHT	KLF10	0	0
Common_E2_OHT	KLF12	0	0
Common_E2_OHT	KLF15	0	0
Common_E2_OHT	KLF17	0	0
Common_E2_OHT	KLF4	0	0
Common_E2_OHT	KLF5	0	0
Common_E2_OHT	KLF7	0	0
Common_E2_OHT	KLF8	0	0
Common_E2_OHT	KLF9	0	0
Common_E2_OHT	MECOM	0	0
Common_E2_OHT	NOBOX	0	0
Common_E2_OHT	OVOL2	0	0
Common_E2_OHT	PEG3	0	0
Common_E2_OHT	PGAM2	0	0
Common_E2_OHT	PRDM13	0	0
Common_E2_OHT	SALL2	0	0
Common_E2_OHT	SALL4	0	0
Common_E2_OHT	SIX1	0	0
Common_E2_OHT	SIX3	0	0
Common_E2_OHT	SIX6	0	0
Common_E2_OHT	TBP	0	0
Common_E2_OHT	TBPL1	0	0
Common_E2_OHT	TBPL2	0	0
Common_E2_OHT	TEAD2	0	0
Common_E2_OHT	TLX2	0	0
Common_E2_OHT	XBP1	0	0
Common_E2_OHT	ZDHHC5	0	0
Common_E2_OHT	ZIC5	0	0
Common_E2_OHT	ZMAT2	0	0
Common_E2_OHT	ZNF148	0	0
Common_E2_OHT	ZNF281	0	0
Common_E2_OHT	ZNF460	0	0
Common_E2_OHT	ZNF740	0	0
Common_E2_OHT	HOXB7	0	-0.586498280850372
Common_E2_OHT	KLF13	0	-0.597317245275044

Common_E2_OHT	KLF2	0	-0.631235538431652
Common_E2_OHT	KLF11	0	-0.752272135404255
E2_only_OHT_only	RCOR1	1.26209403280307	0
E2_only_OHT_only	EHF	0	0
E2_only_OHT_only	ELF1	0	0
E2_only_OHT_only	ELF2	0	0
E2_only_OHT_only	ELF3	0	0
E2_only_OHT_only	ELF4	0	0
E2_only_OHT_only	ELF5	0	0
E2_only_OHT_only	ELK1	0	0
E2_only_OHT_only	ELK3	0	0
E2_only_OHT_only	ELK4	0	0
E2_only_OHT_only	ERF	0	0
E2_only_OHT_only	ERG	0	0
E2_only_OHT_only	ETS1	0	0
E2_only_OHT_only	ETV1	0	0
E2_only_OHT_only	ETV2	0	0
E2_only_OHT_only	ETV3	0	0
E2_only_OHT_only	ETV4	0	0
E2_only_OHT_only	ETV5	0	0
E2_only_OHT_only	ETV6	0	0
E2_only_OHT_only	ETV7	0	0
E2_only_OHT_only	FEV	0	0
E2_only_OHT_only	FGF19	0	0
E2_only_OHT_only	GABPA	0	0
E2_only_OHT_only	GABPB1	0	0
E2_only_OHT_only	JUN	0	0
E2_only_OHT_only	JUND	0	0
E2_only_OHT_only	MYBL2	0	0
E2_only_OHT_only	NFIC	0	0
E2_only_OHT_only	PPARGC1A	0	0
E2_only_OHT_only	PSMD12	0	0
E2_only_OHT_only	RELA	0	0
E2_only_OHT_only	SPI1	0	0
E2_only_OHT_only	SPIB	0	0
E2_only_OHT_only	SRF	0	0
E2_only_OHT_only	TBL1XR1	0	0
E2_only_OHT_only	TCF12	0	0
E2_only_OHT_only	TFAP2C	0	0
E2_only_OHT_only	ZFX	0	0
E2_only_OHT_only	ZFY	0	0
E2_only_OHT_only	GATA2	0	-0.696877350129272
E2_only_OHT_only	ETS2	0	-0.755323206591533
E2_only_OHT_only	FLI1	0	-1.18690661115943
E2_only_CommonE2_OHT	RARG	1.63882465275859	2.1883484431748
E2_only_CommonE2_OHT	VDR	1.32253996493298	1.90530649475913
E2_only_CommonE2_OHT	ESR1	0	0
E2_only_CommonE2_OHT	ESR2	0	0
E2_only_CommonE2_OHT	ESRRB	0	0
E2_only_CommonE2_OHT	ESRRG	0	0

E2_only_CommonE2_OHT	GLI1	0	0
E2_only_CommonE2_OHT	GLI2	0	0
E2_only_CommonE2_OHT	GLI3	0	0
E2_only_CommonE2_OHT	GLIS1	0	0
E2_only_CommonE2_OHT	GLIS2	0	0
E2_only_CommonE2_OHT	GLIS3	0	0
E2_only_CommonE2_OHT	KLF6	0	0
E2_only_CommonE2_OHT	MZF1	0	0
E2_only_CommonE2_OHT	NR2C1	0	0
E2_only_CommonE2_OHT	NR2F1	0	0
E2_only_CommonE2_OHT	PLAGL1	0	0
E2_only_CommonE2_OHT	PRDM16	0	0
E2_only_CommonE2_OHT	RARB	0	0
E2_only_CommonE2_OHT	ZIC1	0	0
E2_only_CommonE2_OHT	ZIC2	0	0
E2_only_CommonE2_OHT	ZIC3	0	0
E2_only_CommonE2_OHT	ZNF202	0	0
E2_only_CommonE2_OHT	NR2F2	0	-0.886391916250244
OHT_only_CommonE2_OHT	EGR3	1.78805085388839	1.7876688311356
OHT_only_CommonE2_OHT	SREBF1	0.591055015872863	0
OHT_only_CommonE2_OHT	EGR1	0	1.1248199410932
OHT_only_CommonE2_OHT	BRF1	0	0
OHT_only_CommonE2_OHT	BRF2	0	0
OHT_only_CommonE2_OHT	CEBPA	0	0
OHT_only_CommonE2_OHT	CEBPE	0	0
OHT_only_CommonE2_OHT	CEBPG	0	0
OHT_only_CommonE2_OHT	EGR4	0	0
OHT_only_CommonE2_OHT	KLF16	0	0
OHT_only_CommonE2_OHT	KLF3	0	0
OHT_only_CommonE2_OHT	NELFE	0	0
OHT_only_CommonE2_OHT	NFYB	0	0
OHT_only_CommonE2_OHT	PATZ1	0	0
OHT_only_CommonE2_OHT	POLR3G	0	0
OHT_only_CommonE2_OHT	RBBP5	0	0
OHT_only_CommonE2_OHT	SMARCA4	0	0
OHT_only_CommonE2_OHT	SP2	0	0
OHT_only_CommonE2_OHT	SP3	0	0
OHT_only_CommonE2_OHT	SP4	0	0
OHT_only_CommonE2_OHT	SREBF2	0	0
OHT_only_CommonE2_OHT	SUPT20H	0	0
OHT_only_CommonE2_OHT	XRCC4	0	0
OHT_only_CommonE2_OHT	ZBTB17	0	0
OHT_only_CommonE2_OHT	ZBTB7B	0	0
OHT_only_CommonE2_OHT	CEBPD	0	-0.872574287095308
OHT_only_CommonE2_OHT	CEBPB	0	-0.878845531863745

Figure 1								
Figure ID	Normality	Test	Post-hoc test	Comparison	Summary	P-value	Sample size	
							Group	n
Figure 1B	YES	two-way ANOVA	Bonferroni's multiple comparisons test	*P* -interaction between Day and Treatment	****	<0.0001	ERα-WT OVX	12
				P Day	****	<0.0001	ERα-WT OVX+E2	10
				P Treatment	****	<0.0001	ERα-AF1^0 OVX	14
							ERα-AF1^0 OVX+E2	14
Figure 1C	YES	two-way ANOVA	Bonferroni's multiple comparisons test	*P* -interaction between Day and Treatment	****	<0.0001	ERα-WT OVX	16
				P Day	****	<0.0001	ERα-WT OVX+E2	12
				P Treatment	****	<0.0001	ERα-AF2^0 OVX	15
							ERα-AF2^0 OVX+E2	16
Figure 1D	NO	Multiple Mann-Whitney	Benjamini, Kriedger and Yekutieli post-test	ERα-WT OVX vs. ERα-WT OVX+E2	**	0.007991	ERα-WT OVX	10
							ERα-WT OVX+E2	15
				ERα-AF1^0 OVX vs. ERα-AF1^0 OVX+E2	ns	0.535979	ERα-AF1^0 OVX	20
							ERα-AF1^0 OVX+E2	24
Figure 1E	YES	two-way ANOVA	Bonferroni's multiple comparisons test	*P* -interaction between Day and Treatment	****	<0.0001	ERα-WT OVX	14
				P Day	****	<0.0001	ERα-WT OVX+E2	10
				P Treatment	****	<0.0001	ERα-C451A OVX	13
							ERα-C451A OVX+E2	14
Figure 1F	YES	two-way ANOVA	Bonferroni's multiple comparisons test	*P* -interaction between Day and Treatment	****	<0.0001	ERα-WT OVX+Dendrimer	14
				P Day	****	<0.0001	ERα-WT OVX+E2	12
				P Treatment	****	<0.0001	ERα-WT OVX+EDC	15
Figure 1G	NO	Kruskal-Wallis	Dunn's multiple comparisons test	ERα-WT OVX+Dendrimer vs. ERα-WT OVX+E2	ns	0.5299	ERα-WT OVX+Dendrimer	25
				ERα-WT OVX+Dendrimer vs. ERα-WT OVX+EDC	*	0.0378	ERα-WT OVX+E2	48
				ERα-WT OVX+E2 vs. ERα-WT OVX+EDC	$$$$	<0.0001	ERα-WT OVX+EDC	38

Figure 2								
Figure ID	Normality	Test	Post-hoc test	Comparison	Summary	P-value	Sample size	
							Group	n
Figure 2B	YES	two-way ANOVA	Bonferroni's multiple comparisons test	*P* -interaction between Day and Treatment	****	<0.0001	ERα-WT OVX	6
				P Day	****	<0.0001	ERα-WT OVX+E2	5
				P Treatment	****	<0.0001	ERα-WT OVX+TAM	8
Figure 2D	YES	two-way ANOVA	Bonferroni's multiple comparisons test	*P* -interaction between Day and Treatment	****	<0.0001	ERα-WT OVX	16
				P Day	****	<0.0001	ERα-WT OVX+E2	6
				P Treatment	****	<0.0001	ERα-WT OVX+TAM d4	22
							ERα-WT OVX+E2+TAM d4	16
Figure 2F	YES	one-way ANOVA	Bonferroni's multiple comparisons test	ERα-WT OVX *versus* ERα-WT OVX+E2	*	0.0353	ERα-WT OVX	3
				ERα-WT OVX *versus* ERα-WT OVX+TAM d4	ns	>0.9999	ERα-WT OVX+E2	4
				ERα-WT OVX *versus* ERα-WT OVX+E2+TAM d4	ns	>0.9999	ERα-WT OVX+TAM d4	3
				ERα-WT OVX+E2 *versus* ERα-WT+TAM d4	$	0.0186	ERα-WT OVX+E2+TAM d4	5
				ERα-WT OVX+E2 *versus* ERα-WT+E2+TAM d4	$$	0.0022		
				ERα-WT OVX+TAM d4 *versus* ERα-WT+E2+TAM d4	ns	>0.9999		
Figure 2G	YES	one-way ANOVA	Bonferroni's multiple comparisons test	ERα-WT OVX *versus* ERα-WT OVX+E2	**	0.0021	ERα-WT OVX	3
				ERα-WT OVX *versus* ERα-WT OVX+TAM d4	***	0.0006	ERα-WT OVX+E2	4
				ERα-WT OVX *versus* ERα-WT OVX+E2+TAM d4	*	0.0235	ERα-WT OVX+TAM d4	3
				ERα-WT OVX+E2 *versus* ERα-WT+TAM d4	ns	0.2041	ERα-WT OVX+E2+TAM d4	5
				ERα-WT OVX+E2 *versus* ERα-WT+E2+TAM d4	ns	0.1486		
				ERα-WT OVX+TAM d4 *versus* ERα-WT+E2+TAM d4	†	0.0251		
Figure 2I	YES	two-way ANOVA	Bonferroni's multiple comparisons test	*P* -interaction between Day and Treatment	*	0.0302	ERα-WT NT	8
				P Day	****	<0.0001	ERα-WT TAM d4	9
				P Treatment	*	0.0477		
Figure 2K	NO	Mann Whitney		ERα-WT NT *versus* ERα-WT TAM d4	*	0.0109	ERα-WT NT	8
							ERα-WT TAM d4	5
Figure 2L	NO	Mann Whitney		ERα-WT NT *versus* ERα-WT TAM d4	ns	0.1274	ERα-WT NT	8
							ERα-WT TAM d4	5

Figure 3

Figure ID	Normality	Test	Post-hoc test	Comparison	Summary	P-value	Sample size	
							Group	n
Figure 3C	NO	Kruskal-Wallis	Dunn's multiple comparisons test	ERα-WT OVX *versus* ERα-WT OVX+E2	*	0.0201	ERα-WT OVX	15
				ERα-WT OVX *versus* ERα-WT OVX+TAM	ns	>0.9999	ERα-WT OVX+E2	18
				ERα-WT OVX *versus* ERα-WT OVX+E2+TAM	**	0.0026	ERα-WT OVX+TAM	15
				ERα-WT OVX+E2 *versus* ERα-WT+TAM	ns	0.1486	ERα-WT OVX+E2+TAM	35
				ERα-WT OVX+E2 *versus* ERα-WT+E2+TAM	$$$$	<0.0001		
				ERα-WT OVX+TAM d4 *versus* ERα-WT+E2+TAM	†††	0.0001		

Figure 4								
Figure ID	Normality	Test	Post-hoc test	Comparison	Summary	P-value	Sample size	
							Group	n
Figure 4G				***FGF2***				
	YES	one-way ANOVA	Bonferroni's multiple comparisons test	DMSO *versus* E2	****	<0.0001	DMSO	6
				DMSO *versus* 4-OHT	**		E2	6
				DMSO *versus* E2+4-OHT	ns	>0.9999	4-OHT	6
				E2 *versus* 4-OHT	$$$$	<0.0001	E2+4-OHT	6
				E2 *versus* E2+4-OHT	$$$$	<0.0001		
				4-OHT *versus* E2+4-OHT	ns	0.0876		
				VEGFA				
	YES	one-way ANOVA	Bonferroni's multiple comparisons test	DMSO *versus* E2	***	<0.0004	DMSO	6
				DMSO *versus* 4-OHT	****	<0.0001	E2	6
				DMSO *versus* E2+4-OHT	****	<0.0001	4-OHT	6
				E2 *versus* 4-OHT	$$$$	<0.0001	E2+4-OHT	6
				E2 *versus* E2+4-OHT	$$$$	<0.0001		
				4-OHT *versus* E2+4-OHT	ns	>0.9999		
				PDGFB				
	YES	one-way ANOVA	Bonferroni's multiple comparisons test	DMSO *versus* E2	ns	>0.9999	DMSO	6
				DMSO *versus* 4-OHT	****	<0.0001	E2	6
				DMSO *versus* E2+4-OHT	****	<0.0001	4-OHT	6
				E2 *versus* 4-OHT	$$$$	<0.0001	E2+4-OHT	6
				E2 *versus* E2+4-OHT	$$$$	<0.0001		
				4-OHT *versus* E2+4-OHT	ns	>0.9999		
				VEGFR2				
	YES	one-way ANOVA	Bonferroni's multiple comparisons test	DMSO *versus* E2	ns	0.0699	DMSO	6
				DMSO *versus* 4-OHT	****	<0.0001	E2	6
				DMSO *versus* E2+4-OHT	****	<0.0001	4-OHT	6
				E2 *versus* 4-OHT	$$$$	<0.0001	E2+4-OHT	6
				E2 *versus* E2+4-OHT	$$$$	<0.0001		
				4-OHT *versus* E2+4-OHT		>0.9999		
				SCUBE2				
	YES	one-way ANOVA	Bonferroni's multiple comparisons test	DMSO *versus* E2	****	<0.0001	DMSO	6
				DMSO *versus* 4-OHT	*	0.0425	E2	6
				DMSO *versus* E2+4-OHT	ns	>0.9999	4-OHT	6
				E2 *versus* 4-OHT	$$$$	<0.0001	E2+4-OHT	6
				E2 *versus* E2+4-OHT	$$$$	<0.0001		
				4-OHT *versus* E2+4-OHT	ns	0.6559		
				ANGPT2				
	YES	one-way ANOVA	Bonferroni's multiple comparisons test	DMSO *versus* E2	****	<0.0001	DMSO	6
				DMSO *versus* 4-OHT	****	<0.0001	E2	6
				DMSO *versus* E2+4-OHT	****	>0.9999	4-OHT	6
				E2 *versus* 4-OHT	$$$$	<0.0001	E2+4-OHT	6
				E2 *versus* E2+4-OHT	$$$$	<0.0001		
				4-OHT *versus* E2+4-OHT	†††	0.0001		
				DLL4				
	YES	one-way ANOVA	Bonferroni's multiple comparisons test	DMSO *versus* E2	****	<0.0001	DMSO	6
				DMSO *versus* 4-OHT	****	<0.0001	E2	6
				DMSO *versus* E2+4-OHT	****	<0.0001	4-OHT	6
				E2 *versus* 4-OHT	$$$	0.0005	E2+4-OHT	6
				E2 *versus* E2+4-OHT	$$$	0.0004		
				4-OHT *versus* E2+4-OHT	ns	>0.9999		
				DLL1				
	YES	one-way ANOVA	Bonferroni's multiple comparisons test	DMSO *versus* E2	ns	>0.9999	DMSO	6
				DMSO *versus* 4-OHT	****	<0.0001	E2	6
				DMSO *versus* E2+4-OHT	****	<0.0001	4-OHT	6
				E2 *versus* 4-OHT	$$$$	<0.0001	E2+4-OHT	6
				E2 *versus* E2+4-OHT	$$$$	<0.0001		
				4-OHT *versus* E2+4-OHT	ns	0.7021		
				NOTCH1				
	YES	one-way ANOVA	Bonferroni's multiple comparisons test	DMSO *versus* E2	ns	>0.9999	DMSO	6
				DMSO *versus* 4-OHT	****	<0.0001	E2	6
				DMSO *versus* E2+4-OHT	****	<0.0001	4-OHT	6
				E2 *versus* 4-OHT	$$$$	<0.0001	E2+4-OHT	6
				E2 *versus* E2+4-OHT	$$$$	<0.0001		
				4-OHT *versus* E2+4-OHT	ns	>0.9999		
				HEYL				
	YES	one-way ANOVA	Bonferroni's multiple comparisons test	DMSO *versus* E2	****	<0.0001	DMSO	6
				DMSO *versus* 4-OHT	ns	>0.9999	E2	6
				DMSO *versus* E2+4-OHT	ns	<0.7732	4-OHT	6
				E2 *versus* 4-OHT	$$$$	<0.0001	E2+4-OHT	6
				E2 *versus* E2+4-OHT	$$$$	<0.0001		
				4-OHT *versus* E2+4-OHT	ns	>0.9999		
				HEY2				
	YES	one-way ANOVA	Bonferroni's multiple comparisons test	DMSO *versus* E2	**	0.0027	DMSO	6
				DMSO *versus* 4-OHT	****	<0.0001	E2	6
				DMSO *versus* E2+4-OHT	****	<0.0001	4-OHT	6
				E2 *versus* 4-OHT	$$$$	<0.0001	E2+4-OHT	6
				E2 *versus* E2+4-OHT	$$$$	<0.0001		
				4-OHT *versus* E2+4-OHT	ns	>0.9999		
				HEY2				
	YES	one-way ANOVA	Bonferroni's multiple comparisons test	DMSO *versus* E2	**	0.0027	DMSO	6
				DMSO *versus* 4-OHT	****	<0.0001	E2	6
				DMSO *versus* E2+4-OHT	****	<0.0001	4-OHT	6
				E2 *versus* 4-OHT	$$$$	<0.0001	E2+4-OHT	6
				E2 *versus* E2+4-OHT	$$$$	<0.0001		
				4-OHT *versus* E2+4-OHT	ns	>0.9999		
				SOX7				
	YES	one-way ANOVA	Bonferroni's multiple comparisons test	DMSO *versus* E2	****	<0.0001	DMSO	6
				DMSO *versus* 4-OHT	****	<0.0001	E2	6
				DMSO *versus* E2+4-OHT	****	<0.0001	4-OHT	6
				E2 *versus* 4-OHT	$$$$	<0.0001	E2+4-OHT	6
				E2 *versus* E2+4-OHT	$$$$	<0.0001		
				4-OHT *versus* E2+4-OHT	ns	>0.9999		

Figure 5								
Figure ID	Normality	Test	Post-hoc test	Comparison	Summary	P-value	Sample size	
							Group	n
Figure 5C				***PFKFB3***				
	YES	one-way ANOVA	Bonferroni's multiple comparisons test	DMSO *versus* E2	****	<0.0001	DMSO	6
				DMSO *versus* 4-OHT	****	<0.0001	E2	6
				DMSO *versus* E2+4-OHT	****	<0.0001	4-OHT	6
				E2 *versus* 4-OHT	$$$$	<0.0001	E2+4-OHT	6
				E2 *versus* E2+4-OHT	$$$$	<0.0001		
				4-OHT *versus* E2+4-OHT	ns	0.0684		
				HK1				
	YES	one-way ANOVA	Bonferroni's multiple comparisons test	DMSO *versus* E2	****	<0.0001	DMSO	6
				DMSO *versus* 4-OHT	****	<0.0001	E2	6
				DMSO *versus* E2+4-OHT	****	<0.0001	4-OHT	6
				E2 *versus* 4-OHT	$$$$	<0.0001	E2+4-OHT	6
				E2 *versus* E2+4-OHT	$$$$	<0.0001		
				4-OHT *versus* E2+4-OHT	ns	>0.9999		
				HK2				
	YES	one-way ANOVA	Bonferroni's multiple comparisons test	DMSO *versus* E2	****	<0.0001	DMSO	6
				DMSO *versus* 4-OHT	ns	0.5053	E2	6
				DMSO *versus* E2+4-OHT	ns	0.1450	4-OHT	6
				E2 *versus* 4-OHT	$$$	0.0005	E2+4-OHT	6
				E2 *versus* E2+4-OHT	$$	0.0021		
				4-OHT *versus* E2+4-OHT	ns	>0.9999		
				ALDH3B1				
	YES	one-way ANOVA	Bonferroni's multiple comparisons test	DMSO *versus* E2	****	<0.0001	DMSO	6
				DMSO *versus* 4-OHT	****	<0.0001	E2	6
				DMSO *versus* E2+4-OHT	****	<0.0001	4-OHT	6
				E2 *versus* 4-OHT	$$$$	<0.0001	E2+4-OHT	6
				E2 *versus* E2+4-OHT	$$$$	<0.0001		
				4-OHT *versus* E2+4-OHT	ns	>0.9999		
				PDK4				
	YES	one-way ANOVA	Bonferroni's multiple comparisons test	DMSO *versus* E2	****	<0.0001	DMSO	6
				DMSO *versus* 4-OHT	****	<0.0001	E2	6
				DMSO *versus* E2+4-OHT	****	<0.0001	4-OHT	6
				E2 *versus* 4-OHT	$$$$	<0.0001	E2+4-OHT	6
				E2 *versus* E2+4-OHT	$$$$	<0.0001		
				4-OHT *versus* E2+4-OHT	ns	0.1927		
				MPC1				
	YES	one-way ANOVA	Bonferroni's multiple comparisons test	DMSO *versus* E2	****	<0.0001	DMSO	6
				DMSO *versus* 4-OHT	ns	>0.9999	E2	6
				DMSO *versus* E2+4-OHT	ns	0.1537	4-OHT	6
				E2 *versus* 4-OHT	$$$$	<0.0001	E2+4-OHT	6
				E2 *versus* E2+4-OHT	$$$$	<0.0001		
				4-OHT *versus* E2+4-OHT	ns	0.2224		
				AACS				
	YES	one-way ANOVA	Bonferroni's multiple comparisons test	DMSO *versus* E2	****	<0.0001	DMSO	6
				DMSO *versus* 4-OHT	****	<0.0001	E2	6
				DMSO *versus* E2+4-OHT	****	<0.0001	4-OHT	6
				E2 *versus* 4-OHT	$$$$	<0.0001	E2+4-OHT	6
				E2 *versus* E2+4-OHT	$$$$	<0.0001		
				4-OHT *versus* E2+4-OHT	ns	>0.9999		
				ACSS1				
	YES	one-way ANOVA	Bonferroni's multiple comparisons test	DMSO *versus* E2	****	<0.0001	DMSO	6
				DMSO *versus* 4-OHT	**	0.0038	E2	6
				DMSO *versus* E2+4-OHT	*	0.0120	4-OHT	6
				E2 *versus* 4-OHT	$$$$	<0.0001	E2+4-OHT	6
				E2 *versus* E2+4-OHT	$$$$	<0.0001		
				4-OHT *versus* E2+4-OHT	ns	>0.9999		
				MCT1				
	YES	one-way ANOVA	Bonferroni's multiple comparisons test	DMSO *versus* E2	****	<0.0001	DMSO	6
				DMSO *versus* 4-OHT	****	<0.0001	E2	6
				DMSO *versus* E2+4-OHT	****	<0.0001	4-OHT	6
				E2 *versus* 4-OHT	$$$$	<0.0001	E2+4-OHT	6
				E2 *versus* E2+4-OHT	$$$$	<0.0001		
				4-OHT *versus* E2+4-OHT	ns	>0.9999		
				GLUT1				
	YES	one-way ANOVA	Bonferroni's multiple comparisons test	DMSO *versus* E2	****	<0.0001	DMSO	6
				DMSO *versus* 4-OHT	ns	0.6814	E2	6
				DMSO *versus* E2+4-OHT	ns	>0.9999	4-OHT	6
				E2 *versus* 4-OHT	$$$$	<0.0001	E2+4-OHT	6
				E2 *versus* E2+4-OHT	$$$$	<0.0001		
				4-OHT *versus* E2+4-OHT	ns	>0.9999		
				GLUT4				
	YES	one-way ANOVA	Bonferroni's multiple comparisons test	DMSO *versus* E2	****	<0.0001	DMSO	6
				DMSO *versus* 4-OHT	****	<0.0001	E2	6
				DMSO *versus* E2+4-OHT	****	<0.0001	4-OHT	6
				E2 *versus* 4-OHT	ns	>0.9999	E2+4-OHT	6
				E2 *versus* E2+4-OHT	$$	0.0051		
				4-OHT *versus* E2+4-OHT	†††	0.0010		

Figure 6								
**Figure ID**	**Normality**	**Test**	**Post-hoc test**	**Comparison**	**Summary**	**P-value**	**Sample size**	
							**Group**	**n**
				NR2F6				
Figure 6D	YES	one-way ANOVA	Bonferroni's multiple comparisons test	DMSO *versus* E2	****	<0.0001	DMSO	6
				DMSO *versus* 4-OHT	****	<0.0001	E2	6
				DMSO *versus* E2+4-OHT	****	<0.0001	4-OHT	6
				E2 *versus* 4-OHT	$$$$	<0.0001	E2+4-OHT	6
				E2 *versus* E2+4-OHT	$$$$	<0.0001		
				4-OHT *versus* E2+4-OHT	ns	>0.9999		
				SMAD1				
	YES	one-way ANOVA	Bonferroni's multiple comparisons test	DMSO *versus* E2	**	0.0046	DMSO	6
				DMSO *versus* 4-OHT	****	<0.0001	E2	6
				DMSO *versus* E2+4-OHT	***	<0.0001	4-OHT	6
				E2 *versus* 4-OHT	$	<0.0001	E2+4-OHT	6
				E2 *versus* E2+4-OHT	ns	<0.0001		
				4-OHT *versus* E2+4-OHT	ns	>0.9999		
				LTF				
	YES	one-way ANOVA	Bonferroni's multiple comparisons test	DMSO *versus* E2	****	<0.0001	DMSO	6
				DMSO *versus* 4-OHT	****	<0.0001	E2	6
				DMSO *versus* E2+4-OHT	****	<0.0001	4-OHT	6
				E2 *versus* 4-OHT	$$$$	<0.0001	E2+4-OHT	6
				E2 *versus* E2+4-OHT	$$$$	<0.0001		
				4-OHT *versus* E2+4-OHT	ns	>0.9999		
				KLF2				
	YES	one-way ANOVA	Bonferroni's multiple comparisons test	DMSO *versus* E2	****	<0.0001	DMSO	6
				DMSO *versus* 4-OHT	****	<0.0001	E2	6
				DMSO *versus* E2+4-OHT	****	<0.0001	4-OHT	6
				E2 *versus* 4-OHT	$$	0.0041	E2+4-OHT	6
				E2 *versus* E2+4-OHT	$	0.0323		
				4-OHT *versus* E2+4-OHT	ns	>0.9999		
				KLF13				
Figure 6H	YES	one-way ANOVA	Bonferroni's multiple comparisons test	DMSO *versus* E2	****	<0.0001	DMSO	6
				DMSO *versus* 4-OHT	****	<0.0001	E2	6
				DMSO *versus* E2+4-OHT	****	<0.0001	4-OHT	6
				E2 *versus* 4-OHT	$$$$	<0.0001	E2+4-OHT	6
				E2 *versus* E2+4-OHT	$$$$	<0.0001		
				4-OHT *versus* E2+4-OHT	ns	0.8496		
				EGR1				
	YES	one-way ANOVA	Bonferroni's multiple comparisons test	DMSO *versus* E2	****	<0.0001	DMSO	6
				DMSO *versus* 4-OHT	****	<0.0001	E2	6
				DMSO *versus* E2+4-OHT	****	<0.0001	4-OHT	6
				E2 *versus* 4-OHT	$$$$	<0.0001	E2+4-OHT	6
				E2 *versus* E2+4-OHT	$$$$	<0.0001		
				4-OHT *versus* E2+4-OHT	ns	0.5786		
				FOXC1				
	NO	Kruskal-Wallis	Dunn's multiple comparisons test	DMSO *versus* E2	**	0.0020	DMSO	6
				DMSO *versus* 4-OHT	ns	>0.9999	E2	6
				DMSO *versus* E2+4-OHT	ns	>0.9999	4-OHT	6
				E2 *versus* 4-OHT	ns	0.0682	E2+4-OHT	6
				E2 *versus* E2+4-OHT	$	0.0423		
				4-OHT *versus* E2+4-OHT	ns	>0.9999		
				HOXB6				
	YES	one-way ANOVA	Bonferroni's multiple comparisons test	DMSO *versus* E2	****	<0.0001	DMSO	6
				DMSO *versus* 4-OHT	****	<0.0001	E2	6
				DMSO *versus* E2+4-OHT	****	<0.0001	4-OHT	6
				E2 *versus* 4-OHT	$$$$	<0.0001	E2+4-OHT	6
				E2 *versus* E2+4-OHT	$$$$	<0.0001		
				4-OHT *versus* E2+4-OHT	ns	0.3984		
				HOXB7				
	YES	one-way ANOVA	Bonferroni's multiple comparisons test	DMSO *versus* E2	****	<0.0001	DMSO	6
				DMSO *versus* 4-OHT	****	<0.0001	E2	6
				DMSO *versus* E2+4-OHT	****	<0.0001	4-OHT	6
				E2 *versus* 4-OHT	$$$$	<0.0001	E2+4-OHT	6
				E2 *versus* E2+4-OHT	$$$$	<0.0001		
				4-OHT *versus* E2+4-OHT	ns	>0.9999		

Appendix S1								
**Figure ID**	**Normality**	**Test**	**Post-hoc test**	**Comparison**	**Summary**	**P-value**	**Sample size**	
							**Group**	**n**
	ESR1							
Figure S1A	NO	Kruskal-Wallis	Dunn's multiple comparisons test	hERαWT DMSO *versus* hERαWT E2	ns	0.8462	hERαWT DMSO	3
				hERαWT DMSO *versus* hERαWT 4-OHT	ns	0.5366	hERαWT E2	3
				hERαWT DMSO *versus* hERαWT E2+4-OHT	*	0.0194	hERαWT 4-OHT	3
				hERαWT E2 *versus* hERαWT 4-OHT	ns	>0.9999	hERαWT E2+4-OHT	3
				hERαWT E2 *versus* hERαWT E2+4-OHT	ns	0.8462		
				hERαWT 4-OHT *versus* hERαWT E2+4-OHT	ns	>0.9999		

Appendix S2								
Figure ID	Normality	Test	Post-hoc test	Comparison	Summary	P-value	Sample size	
							Group hERα	n
Figure S2				***VEGFA***				
	YES	one-way ANOVA	Bonferroni's multiple comparisons test	DMSO *versus* E2	*	0.0206	DMSO	6
				DMSO *versus* 4-OHT	ns	>0.9999	E2	6
				DMSO *versus* E2+4-OHT	ns	>0.9999	4-OHT	6
				E2 *versus* 4-OHT	$$$	0.0009	E2+4-OHT	6
				E2 *versus* E2+4-OHT	$	0.0451		
				4-OHT *versus* E2+4-OHT	ns	>0.9999		
				VEGFR2				
	YES	one-way ANOVA	Bonferroni's multiple comparisons test	DMSO *versus* E2	ns	0.6518	DMSO	6
				DMSO *versus* 4-OHT	****	<0.0001	E2	6
				DMSO *versus* E2+4-OHT	*	<0.0001	4-OHT	6
				E2 *versus* 4-OHT	$$	0.0016	E2+4-OHT	6
				E2 *versus* E2+4-OHT	$$	0.0043		
				4-OHT *versus* E2+4-OHT	ns	>0.9999		
				DLL4				
	YES	one-way ANOVA	Bonferroni's multiple comparisons test	DMSO *versus* E2	****	<0.0001	DMSO	6
				DMSO *versus* 4-OHT	****	<0.0001	E2	6
				DMSO *versus* E2+4-OHT	****	<0.0001	4-OHT	6
				E2 *versus* 4-OHT	ns	>0.9999	E2+4-OHT	6
				E2 *versus* E2+4-OHT	ns	>0.9999		
				4-OHT *versus* E2+4-OHT	ns	>0.9999		
				HEY2				
	YES	one-way ANOVA	Bonferroni's multiple comparisons test	DMSO *versus* E2	**	0.0019	DMSO	6
				DMSO *versus* 4-OHT	****	<0.0001	E2	6
				DMSO *versus* E2+4-OHT	****	<0.0001	4-OHT	6
				E2 *versus* 4-OHT	$$$$	<0.0001	E2+4-OHT	6
				E2 *versus* E2+4-OHT	$$$$	<0.0001		
				4-OHT *versus* E2+4-OHT	ns	>0.9999		
				PFKFB3				
	YES	one-way ANOVA	Bonferroni's multiple comparisons test	DMSO *versus* E2	**	0.0065	DMSO	6
				DMSO *versus* 4-OHT	ns	>0.9999	E2	6
				DMSO *versus* E2+4-OHT	ns	>0.9999	4-OHT	6
				E2 *versus* 4-OHT	$$	0.0019	E2+4-OHT	6
				E2 *versus* E2+4-OHT	$$	0.0047		
				4-OHT *versus* E2+4-OHT	ns	>0.9999		
				GLUT1				
	YES	one-way ANOVA	Bonferroni's multiple comparisons test	DMSO *versus* E2	****	<0.0001	DMSO	6
				DMSO *versus* 4-OHT	****	<0.0001	E2	6
				DMSO *versus* E2+4-OHT	****	<0.0001	4-OHT	6
				E2 *versus* 4-OHT	ns	>0.9999	E2+4-OHT	6
				E2 *versus* E2+4-OHT	ns	>0.9999		
				4-OHT *versus* E2+4-OHT	ns	>0.9999		
				PDK4				
	YES	one-way ANOVA	Bonferroni's multiple comparisons test	DMSO *versus* E2	****	<0.0001	DMSO	6
				DMSO *versus* 4-OHT	ns	>0.9999	E2	6
				DMSO *versus* E2+4-OHT	ns	0.0595	4-OHT	6
				E2 *versus* 4-OHT	$$$$	<0.0001	E2+4-OHT	6
				E2 *versus* E2+4-OHT	$$$$	<0.0001		
				4-OHT *versus* E2+4-OHT	ns	0.5497		
				MPC1				
	YES	one-way ANOVA	Bonferroni's multiple comparisons test	DMSO *versus* E2	****	<0.0001	DMSO	6
				DMSO *versus* 4-OHT	ns	>0.9999	E2	6
				DMSO *versus* E2+4-OHT	ns	>0.9999	4-OHT	6
				E2 *versus* 4-OHT	$$$$	<0.0001	E2+4-OHT	6
				E2 *versus* E2+4-OHT	$$$$	<0.0001		
				4-OHT *versus* E2+4-OHT	ns	>0.9999		

Appendix S3

Figure ID	Normality	Test	Post-hoc test	Comparison	Summary	P-value	Sample size<br>Group NT	n	Sample size<br>Group hERα	n
				NR2F6						
	YES	two-way ANOVA	Bonferroni's multiple comparisons test	*P* -interaction between Genotype and Treatment	<0.0001	****	DMSO	5	DMSO	6
				P Genotype	<0.0001	****	E2	6	E2	6
				P Treatment	<0.0001	****	4-OHT	5	4-OHT	6
							E2+4-OHT	6	E2+4-OHT	6
				ETS2						
	YES	two-way ANOVA	Bonferroni's multiple comparisons test	*P* -interaction between Genotype and Treatment	0.0137	*	DMSO	5	DMSO	6
				P Genotype	0.0223	*	E2	6	E2	6
				P Treatment	<0.0001	****	4-OHT	5	4-OHT	6
							E2+4-OHT	6	E2+4-OHT	6
				SMAD1						
	YES	two-way ANOVA	Bonferroni's multiple comparisons test	*P* -interaction between Genotype and Treatment	0.2305	ns	DMSO	5	DMSO	6
				P Genotype	0.3293	ns	E2	6	E2	6
				P Treatment	<0.0001	****	4-OHT	5	4-OHT	6
							E2+4-OHT	6	E2+4-OHT	6
Figure S3										
				NR2F2						
	YES	two-way ANOVA	Bonferroni's multiple comparisons test	*P* -interaction between Genotype and Treatment	0.1561	ns	DMSO	5	DMSO	6
				P Genotype	<0.0001	****	E2	6	E2	6
				P Treatment	0.0039	**	4-OHT	5	4-OHT	6
							E2+4-OHT	6	E2+4-OHT	6
				SMAD6						
	YES	two-way ANOVA	Bonferroni's multiple comparisons test	*P* -interaction between Genotype and Treatment	0.6219	ns	DMSO	5	DMSO	6
				P Genotype	<0.0001	****	E2	6	E2	6
				P Treatment	<0.0001	****	4-OHT	5	4-OHT	6
							E2+4-OHT	6	E2+4-OHT	6
				FOXC1						
	YES	two-way ANOVA	Bonferroni's multiple comparisons test	*P* -interaction between Genotype and Treatment	<0.0001	****	DMSO	5	DMSO	6
				P Genotype	<0.0001	****	E2	6	E2	6
				P Treatment	<0.0001	****	4-OHT	5	4-OHT	6
							E2+4-OHT	6	E2+4-OHT	6
